# Brown adipocyte-specific knockout of Bmal1 causes mild but significant thermogenesis impairment in mice

**DOI:** 10.1016/j.molmet.2021.101202

**Published:** 2021-03-03

**Authors:** Nazmul Hasan, Naoto Nagata, Jun-ichi Morishige, Md Tarikul Islam, Zheng Jing, Ken-ichi Harada, Michihiro Mieda, Masanori Ono, Hiroshi Fujiwara, Takiko Daikoku, Tomoko Fujiwara, Yoshiko Maida, Tsuguhito Ota, Shigeki Shimba, Shuichi Kaneko, Akio Fujimura, Hitoshi Ando

**Affiliations:** 1 Department of Cellular and Molecular Function Analysis, Graduate School of Medical Sciences, Kanazawa University, Kanazawa, Japan; 2 Department of Integrative Neurophysiology, Graduate School of Medical Sciences, Kanazawa University, Kanazawa, Japan; 3 Department of Human Pathology, Graduate School of Medical Sciences, Kanazawa University, Kanazawa, Japan; 4 Department of Obstetrics and Gynecology, Graduate School of Medical Sciences, Kanazawa University, Kanazawa, Japan; 5 Institute for Experimental Animals, Advanced Science Research Center, Kanazawa University, Kanazawa, Japan; 6 Department of Social Work and Life Design, Kyoto Notre Dame University, Kyoto, Japan; 7 Department of Health Development Nursing, Graduate School of Medical Sciences, Kanazawa University, Kanazawa, Japan; 8 Department of Internal Medicine, Fukui-ken Saiseikai Hospital, Fukui, Japan; 9 Department of Health Science, School of Pharmacy, Nihon University, Funabashi, Japan; 10 Department of Gastroenterology, Graduate School of Medical Sciences, Kanazawa University, Kanazawa, Japan; 11 Department of Pharmacology, School of Medicine, Jichi Medical University, Shimotsuke, Japan

**Keywords:** Brown adipose tissue, Circadian rhythm, Clock genes, Fatty acids, Obesity, Thermogenesis, BAT, Brown adipose tissue, BA-Bmal1 KO, Brown adipocyte-specific Bmal1 knockout, KO, Knockout, SNS, Sympathetic nervous system, UCP1, Uncoupling protein 1, ZT, Zeitgeber time

## Abstract

**Objective:**

Impaired circadian clocks can cause obesity, but their pathophysiological role in brown adipose tissue (BAT), a major tissue regulating energy metabolism, remains unclear. To address this issue, we investigated the effects of complete disruption of the BAT clock on thermogenesis and energy expenditure.

**Methods:**

Mice with brown adipocyte-specific knockout of the core clock gene *Bmal1* (BA-Bmal1 KO) were generated and analyzed.

**Results:**

The BA-Bmal1 KO mice maintained normal core body temperatures by increasing shivering and locomotor activity despite the elevated expression of thermogenic uncoupling protein 1 in BAT. BA-Bmal1 KO disrupted 24 h rhythmicity of fatty acid utilization in BAT and mildly reduced both BAT thermogenesis and whole-body energy expenditure. The impact of BA-Bmal1 KO on the development of obesity became obvious when the mice were fed a high-fat diet.

**Conclusions:**

These results reveal the importance of the BAT clock for maintaining energy homeostasis and preventing obesity.

## Highlights

• The circadian clock in brown adipocytes regulates the rhythmicity of lipid utilization.
• Disruption of the brown adipocyte clock impairs thermogenesis.
• Muscle thermogenesis somewhat compensates for impaired brown adipocyte function.
• BA-Bmal1 KO mice are more prone to diet-induced obesity.

## Graphical abstract

**Figure:**
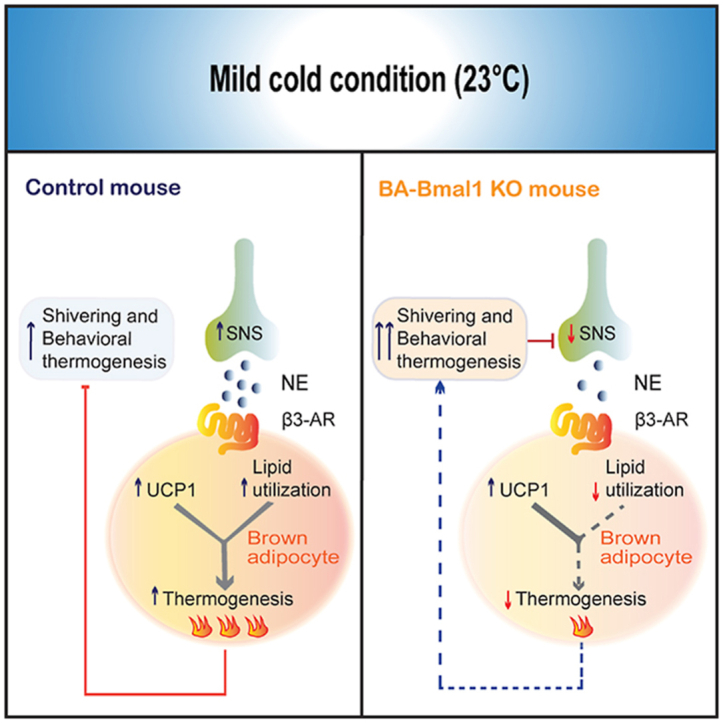

## 1 Introduction

Obesity and associated comorbidities, including type 2 diabetes and cardiovascular diseases, are major worldwide health concerns. Adaptive thermogenesis, which is defined as the regulated production of heat in response to environmental changes in temperature and diet, can be utilized to counteract the hypercaloric state of obesity [1]. There are two forms of adaptive thermogenesis, shivering and non-shivering thermogenesis, and brown adipose tissue (BAT) is the major site of non-shivering thermogenesis. BAT's activity is associated with obesity in humans [2]. Moreover, genetic ablation of either BAT or uncoupling protein 1 (UCP1), the protein responsible for the thermogenic process in BAT, predisposes mice to obesity [3,4]. At least in mice, induction of UCP1 by treatment with a β3-adrenaergic agonist increases both BAT thermogenesis and whole-body oxygen consumption, preventing the development of obesity [5].

The molecular circadian clock, which is primarily composed of transcriptional/translational feedback loops involving a set of clock genes, resides in almost all cell types [6]. In mammals, the central clock localized in the hypothalamic suprachiasmatic nuclei is regulated by light stimuli, whereas the peripheral clocks located in other tissues may be entrained by a combination of various humoral and neural signals regulated, at least partly, by the central clock [7]. Accumulating evidence has suggested a link between circadian clocks and obesity-related diseases. Chronic jet lag under abnormal lighting conditions, which cause the systemic disruption of circadian clocks, reportedly causes obesity in both mice [8] and shift workers [9]. The mutation of *Clock*, one of the core clock genes, induces obesity and metabolic syndrome in mice [10], and this *Clock* mutant is unable to maintain their body temperature following 12 h of fasting [11], suggesting impaired non-shivering thermogenesis. In humans, genetic variants of *CLOCK* and another core clock gene *BMAL1* are associated with susceptibility to obesity and type 2 diabetes, respectively [12,13]. Peripheral clocks are also impaired in obese diabetic mice [14,15] and patients with type 2 diabetes [16].

The pathophysiological roles of circadian clocks in each peripheral tissue are gradually becoming clear. For example, the clock genes *Bmal1* and *Dbp* are required for adipose differentiation in preadipocytes and lipogenesis in adipocytes [17,18]. Pancreas-specific *Bmal1* knockout (KO) mice develop diabetes due to impaired insulin secretion [19] because the circadian clock in pancreatic β cells plays a role in insulin exocytosis [20]. Dyar et al. [21] demonstrated that the muscle clock controls glucose uptake and metabolism, and our group [22] revealed that the hepatic clock regulates the daily rhythms of gluconeogenesis and fasting glucose levels using muscle- and liver-specific *Bmal1* KO mice, respectively. Regarding BAT, systemic KO of the clock gene *Nr1d1* (also known as *Rev-erb α*) in mice abolishes normal daily rhythms of BAT activity and body temperature and increases cold tolerance because NR1D1 is a major negative transcriptional regulator of not only *Bmal1* but also *Ucp1* [23]. However, mice harboring a global mutation in the clock gene *Per2* are more sensitive to cold because PER2 may modulate UCP1 expression as a co-activator of peroxisome proliferator-activated receptor alpha (PPARα) [24]. Thus, the effect of each clock gene on BAT activity is diverse, and the pathophysiological role of the whole circadian clock in BAT remains unclear.

To address this issue, we generated mice with brown adipocyte-specific KO of the main core clock gene *Bmal1* (BA-Bmal1 KO) and investigated the effects of complete disruption of the BAT clock on thermogenesis and energy expenditure.

## 2 Materials and methods

### 2.1 Animals

To generate BA-Bmal1 KO mice, *Bmal1*-floxed mice [25] were crossed with mice expressing a *Cre* transgene driven by the *Ucp1* promoter (stock no. 024670, Jackson Laboratory, Bar Harbor, ME, USA) [26]. The mice were maintained under controlled temperature (∼23 °C), humidity (∼55%), and light (12-h/12-h light/dark cycle) conditions and fed a regular diet (CRF-1, Oriental Yeast, Tokyo, Japan) and water ad libitum. A subset of mice was fed a high-fat diet (D12492, Research Diets, New Brunswick, NJ, USA) for 22 weeks. Male mice (8–9 weeks old [unless specified]) were used for the experiments, and *Bmal1*-floxed mice were used as controls. All of the animal procedures were approved by the Institutional Committee for Ethical Use of Experimental Animals (approval no. AP-173889) and performed in accordance with the Guidelines for the Care and Use of Laboratory Animals at Kanazawa University (Kanazawa, Japan).

### 2.2 RNA isolation and quantitative PCR

The mice were sacrificed to obtain blood, interscapular BAT, and epididymal fat samples every 4 h for 24 h. Total RNA was extracted from frozen tissues by TRIzol using an RNeasy Mini Kit (Qiagen, Valencia, CA, USA), and cDNA was synthesized using a High-Capacity cDNA Reverse Transcription Kit (Thermo Fisher Scientific, Waltham, MA, USA) according to the manufacturers’ instructions. Gene expression was analyzed by quantitative PCR performed using an Applied Biosystems ViiA 7 Real-Time PCR System. The specific sets of primers and TaqMan probes (TaqMan gene expression assays) used in this study are listed in Supplemental Table 1. Data were analyzed using the comparative threshold cycle method with *Rplp0* as the internal control.

### 2.3 Western blotting analyses

Total protein lysates were prepared from frozen tissues by homogenization in RIPA lysis buffer containing protease inhibitor cocktail (Nacalai Tesque, Kyoto, Japan), and subjected to sodium dodecyl sulfate polyacrylamide gel electrophoresis. The resolved proteins were transferred to PVDF membranes. The membranes were incubated overnight at 4 °C with primary antibodies and then incubated with appropriate secondary antibodies. The antibodies are listed in the Supplemental Key resources table. Protein bands were imaged using an ImageQuant LAS 4000 Camera System (GE Healthcare, Chicago, IL, USA) after visualization with EzWestLumi Plus (ATTO, Tokyo, Japan), and the pixel density was quantified with Image Studio Lite software version 5.2 (LI-COR, Lincoln, NE, USA).

### 2.4 Measurements of glucose and lipid concentrations

Blood glucose levels were determined in tail blood using a Glucocard G+ meter (Arkray, Kyoto, Japan). Serum concentrations of total cholesterol, triglycerides, and non-esterified fatty acids were measured using commercial kits (LabAssay, Fujifilm Wako Pure Chemical, Osaka, Japan).

### 2.5 Core body temperature and locomotor activity

At 7 weeks of age, the mice were implanted intraperitoneally with an ultra-small temperature logger (DST Nano-T, Star-Oddi, Gardabaer, Iceland) under anesthesia and housed individually in specialized cages for an infrared sensor detection system (Supermex, Muromachi Kikai, Tokyo, Japan). Two weeks later, core body temperature and locomotor activity were continuously measured for five consecutive days.

### 2.6 Electroencephalography and electromyography

Implantation of electroencephalography/electromyography (EEG/EMG) electrodes, EEG/EMG recordings, and data analyses were performed as previously described [27]. Specifically, all of the mice were provided at least 14 days to recover from surgery prior to the start of EEG/EMG recording. Before starting the EEG/EMG recording, the mice were habituated in the recording chamber for at least 3 days. EEG/EMG signals were amplified through an amplifier (MEG-6108, Nihon Kohden, Tokyo, Japan) and recorded on a computer using recording software (Vital Recorder, Kissei Comtec, Matsumoto, Japan). The polysomnographic recordings were scored using sleep analysis software (Sleep Sign, Kissei Comtec). Scoring was executed based on visual inspection of the EEG and EMG waveforms as well as the power spectra of 4 s epochs. We defined wakefulness by the presence of desynchronized low-amplitude EEG activity and an increase in EMG activity. We scored non-rapid eye movement (non-REM) sleep when EEG showed high-amplitude and low-frequency (0.5–4 Hz) oscillations along with reduced EMG activity. We defined REM sleep by the presence of theta rhythms (4–10 Hz) and a very low muscle tone.

For EMG data analyses, we selected the first four time frames per hour of the non-REM sleep period when the waveform was stable with no high-amplitude spike for at least for 16 s. Then the raw EMG signal was rectified using Sleep Sign software (Kissei Comtec) amplified 1,000 times, and the root mean square (RMS) of the EMG signal was calculated.

### 2.7 Thermographic imaging

The dorsal side of the mice was shaved 48 h before taking images, and thereafter the mice were kept individually. Thermal images (at least 3 images/mouse) were taken from non-anesthetized mice at ZT 8 using a FLIR C2 thermal camera (FLIR Systems, Wilsonville, OR, USA) with an emissivity of 0.97 and a 0.25 m distance. Images were analyzed using FLIR Tools software. The highest temperature values of the selected regions (interscapular and dorsal region) were retrieved and subjected to analyses of the ratio of BAT to the dorsal region.

### 2.8 Measurement of urinary catecholamines

The mice were placed individually in metabolic cages with free access to food and water. After 4 days of acclimation, 24 h urine samples were collected in glass vials containing 60 μL of 6 M hydrochloric acid [28] for four consecutive days. The concentrations of epinephrine, norepinephrine, and dopamine were measured using the established method of high-performance liquid chromatography with post-column fluorescence derivatization [29].

### 2.9 Histological examination

BAT was fixed in 4% paraformaldehyde phosphate-buffered solution (Nacalai Tesque), embedded in paraffin, and stained with hematoxylin and eosin. Analyses of the fat area were conducted using QuPath software [30] in a blinded fashion.

### 2.10 Indirect calorimetry measurements

The mice were individually housed in chambers of an Oxymax system (Columbus Instruments, Columbus, OH, USA). After a 2-day acclimation period, their oxygen consumption and carbon dioxide production were measured for six consecutive days. The respiratory exchange ratio (RER) and energy expenditure (heat) were computed using standard equations.

### 2.11 Metabolome analysis

Approximately 40 mg of frozen tissue was homogenized in 50% (v/v) acetonitrile/water containing internal standards (H3304-1002, Human Metabolome Technologies [HMT], Tsuruoka, Yamagata, Japan). The homogenate was then centrifuged, and the upper aqueous layer was centrifugally filtered through a 5-kDa cutoff filter (Ultrafree MC-PLHCC, HMT) at 9,100×*g* and 4 °C for 120 min to remove macromolecules. The filtrate was evaporated to dryness under a vacuum and reconstituted in Milli-Q water for metabolome analysis at HMT.

Metabolome analysis was conducted via the HMT C-SCOPE package using capillary electrophoresis time-of-flight mass spectrometry (CE-TOFMS) for cation analysis and CE-tandem mass spectrometry (CE-MS/MS) for anion analysis based on previously described methods [31,32]. Briefly, CE-TOFMS and CE-MS/MS analysis were carried out using an Agilent CE capillary electrophoresis system (Agilent Technologies, Santa Clara, CA, USA) equipped with an Agilent 6210 time-of-flight mass spectrometer and Agilent 6460 Triple Quadrupole LC/MS, respectively. The systems were controlled by Agilent G2201AA ChemStation software version B.03.01 for CE and connected by a fused silica capillary (50 μm i.d. × 80 cm total length) with commercial electrophoresis buffer (H3301-1001 and I3302-1023 for cation and anion analyses, respectively, HMT) as the electrolyte. The time-of-flight mass spectrometer was scanned from *m*/*z* 50 to 1,000 and the triple quadrupole mass spectrometer was used to detect compounds in the dynamic MRM mode. Peaks were extracted using MasterHands automatic integration software (Keio University, Tsuruoka, Yamagata, Japan) [33] and MassHunter Quantitative Analysis B.04.00 (Agilent Technologies) to obtain peak information including the *m*/*z*, peak area, and migration time (MT). Signal peaks were annotated according to the HMT metabolite database based on their *m*/*z* values with the MTs. The peak area of each metabolite was normalized with respect to the internal standard area, and the metabolite concentration was evaluated by standard curves with three-point calibrations using each standard compound. Hierarchical cluster analysis and principal component analysis [34] were performed by HMT's proprietary MATLAB and R programs, respectively. Detected metabolites were plotted on metabolic pathway maps using VANTED software [35].

### 2.12 Statistical analysis

Data are presented as the mean and standard deviation (SD). Differences between the genotypes were analyzed using Student's t test or the Mann–Whitney U test. Regression analyses were conducted to assess the relationship between locomotor activity and core body temperature. The calculations were performed using IBM SPSS Statistics (version 24.0). P < 0.05 was considered statistically significant.

## 3 Results

### 3.1 BA-Bmal1 KO increased UCP1 expression in BAT

**Figure 1:**
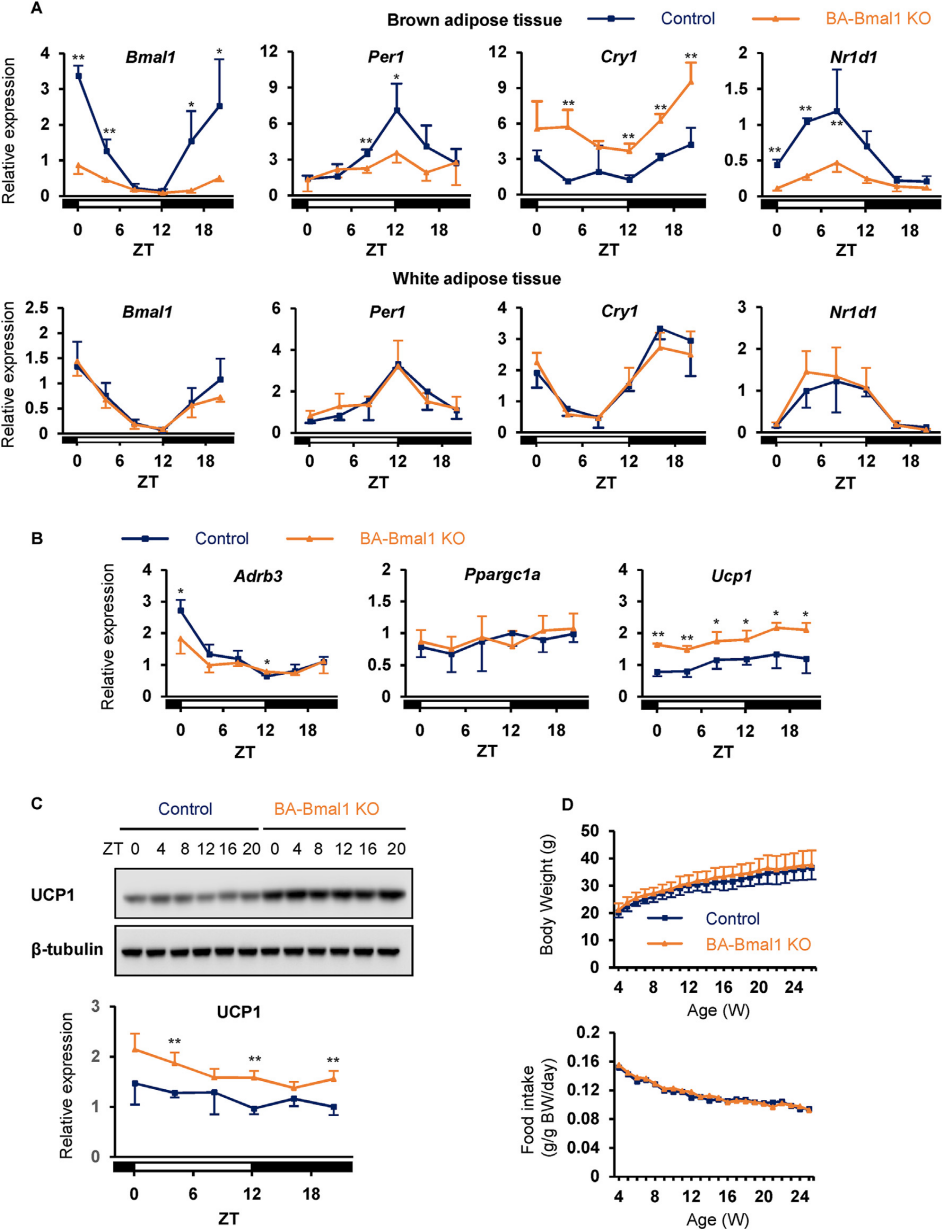
**BA-Bmal1 KO increased UCP1 expression in BAT.** (A–C) Daily expression profiles of the clock genes and thermogenesis-related genes. The mice housed at 23 °C were sacrificed to obtain brown (A–C) and white adipose tissue samples (A) at the following ZTs: 0, 4, 8, 12, 16, and 20, in which ZT 0 was defined as lights on and ZT 12 as lights off. The mRNA expression of the clock genes (A) and thermogenesis-related genes (B) and protein expression of UCP1 (C) were quantified using quantitative PCR and Western blotting analyses, respectively. Data are presented as the mean and SD of 3–4 mice per time point per group. ∗p < 0.05 and ∗∗p < 0.01 at each time point. (D) Body weight and food intake during normal chow feeding. The mice were housed three animals per cage. Data are presented as the mean and SD of six mice or means of two cages.

The BA-Bmal1 KO mice were generated by crossing *Bmal1*-floxed mice [22,25] with *Ucp1*-Cre mice [26]. As expected, the daily rhythmic mRNA expression levels of *Bmal1* were extensively dampened in the BAT but not in the white adipose tissue of the BA-Bmal1 KO mice (Figure 1A). Consistent with this, the rhythmic mRNA expression of other clock genes (*Per1*, *Cry1*, and *Nr1d1*) was also markedly altered in the BAT. Probably because NR1D1 acts as a repressor of *Cry1* [36], the mRNA levels of *Cry1* were significantly elevated.

The thermogenic activity of BAT is mediated by the sympathetic nervous system (SNS) through the action of the β3-adrenergic receptor (encoded by *Adrb3*), which ultimately activates the transcription of *Ucp1* partly due to the increase in PPARγ coactivator-1α (encoded by *Ppargc1a*) [37]. In the BAT of the controls (*Bmal1*-floxed mice), the mRNA expression of *Adrb3* exhibited an obvious 24 h rhythm with a peak at the beginning of the light phase (zeitgeber time [ZT] 0; ZT was used to describe the experimental time with ZT 0 defined as lights on and ZT 12 as lights off) (Figure 1B). This rhythm was dampened in the BA-Bmal1 KO mice. Although the mRNA levels of *Ppargc1a* did not differ between genotypes, both mRNA and protein levels of UCP1 were significantly elevated for 24 h in the BA-Bmal1 KO mice as reported in systemic *Nr1d1* KO mice [23] (Figure 1B,C). However, this increase in UCP1 did not affect the body weight, food intake, body composition, and circulating glucose and lipid concentrations under a normal chow diet (Figure 1D and Table 1).

**Table 1:** Body weight, circulating glucose and lipid concentrations, and body composition of normal chow (NC) and high-fat diet (HFD)-fed mice.

		NC (8 weeks of age)		HFD (30 weeks of age)	
		Control	BA-Bmal1 KO	Control	BA-Bmal1 KO

*n*		4	5	3	9
Body weight (g)		24.2 ± 1.1	22.6 ± 2.5	46.7 ± 8.6	58.7 ± 3.5∗∗
Blood glucose (mg/dl)		152 ± 10	152 ± 12	188 ± 27	184 ± 21
Serum lipid concentration	TC (mg/dl)	149 ± 24	132 ± 16	426 ± 103	440 ± 67
	TG (mg/dl)	136 ± 23	114 ± 27	89 ± 10	82 ± 15
	NEFA (mEq/l)	1.10 ± 0.26	0.96 ± 0.17	1.01 ± 0.21	0.89 ± 0.25
Tissue weight (g)	iBAT	0.06 ± 0.01	0.06 ± 0.01	0.36 ± 0.24	0.70 ± 0.22∗
	ingWAT	0.22 ± 0.01	0.19 ± 0.03	2.54 ± 0.67	3.26 ± 0.34∗
	eWAT	0.36 ± 0.06	0.30 ± 0.07	1.53 ± 0.69	1.17 ± 0.26
	asWAT	0.30 ± 0.03	0.25 ± 0.06	1.57 ± 0.38	3.68 ± 0.55∗∗
	prWAT	0.06 ± 0.01	0.04 ± 0.01	0.92 ± 0.16	1.62 ± 0.18∗∗
	Liver	1.15 ± 0.08	1.02 ± 0.11	2.97 ± 1.37	3.70 ± 0.57

Values are presented as the mean ± SD. ∗p < 0.05 and ∗∗p < 0.01 vs control. TC, total cholesterol; TG, triglycerides; NEFA, non-esterified fatty acid; iBAT, interscapular brown adipose tissue; ingWAT, inguinal white adipose tissue; eWAT, epididymal white adipose tissue; asWAT, anterior subcutaneous white adipose tissue; prWAT, perirenal white adipose tissue.

### 3.2 BA-Bmal1 KO increased locomotor activity without affecting core body temperature

**Figure 2:**
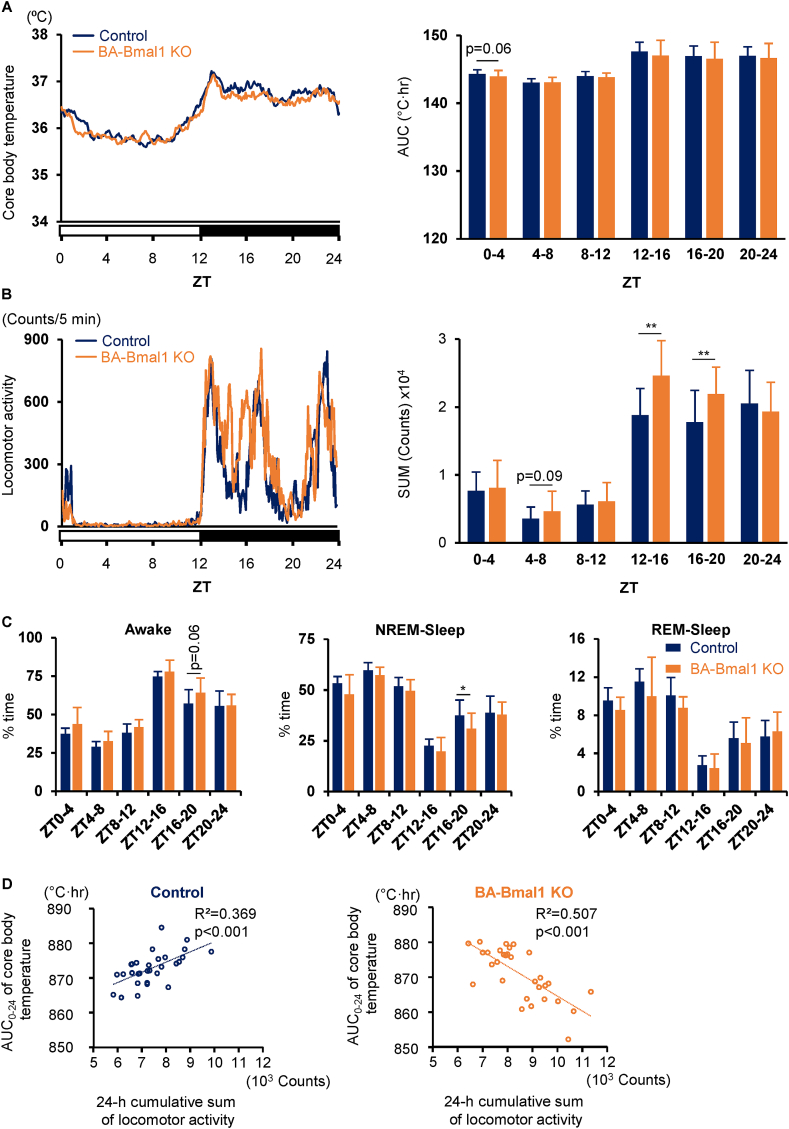
**BA-Bmal1 KO increased locomotor activity without affecting core body temperature.** (A and B) Daily profiles of core body temperature (A) and locomotor activity (B). AUC, area under the curve. Data are presented as the mean (left) or mean +SD (right) of 30 values obtained from six mice. ∗∗p < 0.01. (C) Daily sleep-wake rhythm. Data are presented as the mean +SD of six mice. ∗p < 0.05. (D) Association between core body temperature and locomotor activity.

The themogenic activity of the BAT, which greatly depends on the UCP1 expression, plays an important role in maintaining body temperature [1]. Therefore, we investigated the effects of BA-Bmal1 KO on the core body temperature. The BA-Bmal1 KO mice housed at 23 °C, a mild cold temperature for rodents, showed a similar 24 h core body temperature rhythm compared to the control mice (Figure 2A). The body temperature tended to be lower during the early rest phase (ZT 0–4) in the BA-Bmal1 KO mice than in the controls. Moreover, the locomotor activity significantly increased, particularly in the active phase (Figure 2B). Consistently, the duration of non-rapid eye movement sleep was shorter in the middle of the active phase (ZT 16–20) in the BA-Bmal1 KO mice (Figure 2C). Interestingly, the 24 h area under the core body temperature time curve was positively correlated with the 24 h cumulative sum of locomotor activity in the control mice, but the correlation was completely inverted in the BA-Bma11 KO mice (Figure 2D). Namely, the lower the body temperature, the higher the activity in the BA-Bmal1 KO strain. Because increased locomotor activity is one behavioral strategy for maintaining body temperature [38], these results suggest that BA-Bmal1 KO increases behavioral thermogenesis and raise the possibility that BA-Bmal1 KO does not enhance but rather reduces thermogenesis despite the increased expression of UCP1 in the BAT.

### 3.3 BA-Bmal1 KO mildly reduced thermogenesis in BAT

**Figure 3:**
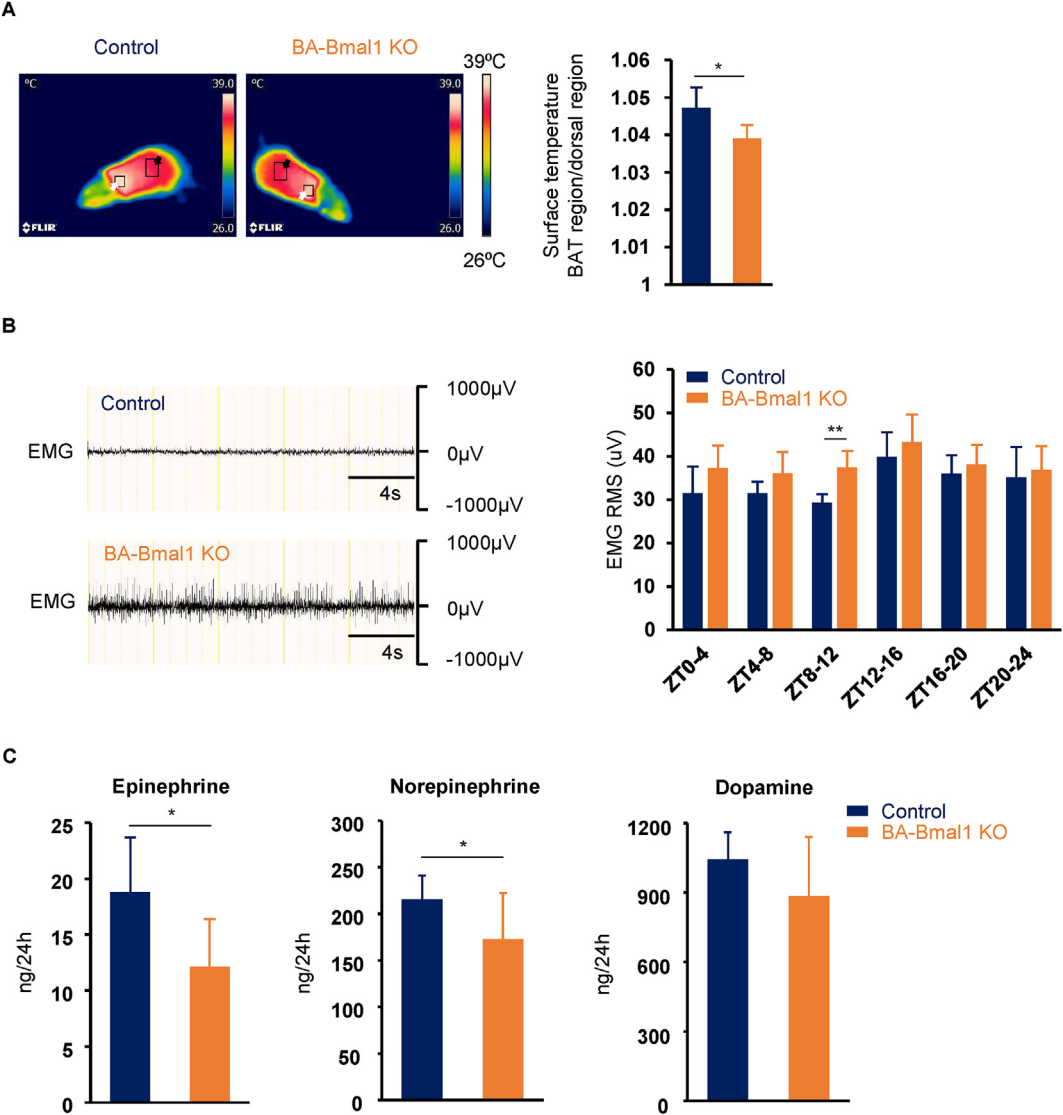
**BA-Bmal1 KO mildly reduced thermogenesis in BAT.** (A) Representative thermal images (left) and the temperature ratio of the interscapular BAT region to the dorsal region (right; n = 5, each group), which were taken at ZT 8. The box indicated by the white arrow specifies the interscapular BAT region, and the box indicated by the black arrow specifies the dorsal region. The highest temperature values of these selected regions were retrieved and analyzed. ∗p < 0.05. (B) Representative EMG signals (left) and daily EMG RMS profiles (right; n = 6, each group). ∗∗p < 0.01. (C) Urinary excretion of catecholamines. Data are presented as the mean +SD of 20 values obtained from five mice. ∗p < 0.05.

To determine if the BA-Bmal1 KO mice had reduced thermogenesis in the BAT, we compared the surface temperature of the interscapular BAT region between the BA-Bmal1 KO and their littermate control mice. Although the BAT surface temperature itself did not differ between genotypes (37.4 ± 0.7 °C in the control mice vs 38.0 ± 0.4 °C in the BA-Bmal1 KO mice; p = 0.16), we found that the temperature ratio of the BAT region to dorsal region, which was measured at ZT 8, was significantly lower in the BA-Bmal1 KO mice than in the control mice (Figure 3A). Consistent with this, the shivering intensity increased at least during the late rest phase (ZT 8–12) (Figure 3B). Taken together, these findings indicate that both shivering and behavioral thermogenesis, which are not mediated by the SNS [39], adequately compensated the impaired non-shivering thermogenesis in the BAT to maintain the core body temperature. Possibly as a result, the SNS activity assessed by daily urinary excretion of catecholamines decreased in the BA-Bmal1 KO mice (Figure 3C). It is noteworthy, however, that most of the observed effects of BA-Bmal1 KO were temporal and relatively mild. In fact, the tolerance to cold exposure (4 °C) of the BA-Bmal1 KO mice was comparable to that of the control mice (Supplemental Fig. 1). Thus, the impairment in BAT function in the BA-Bmal1 KO mice seemed to be very mild.

### 3.4 BA-Bmal1 KO mildly impaired lipid utilization in BAT

**Figure 4:**
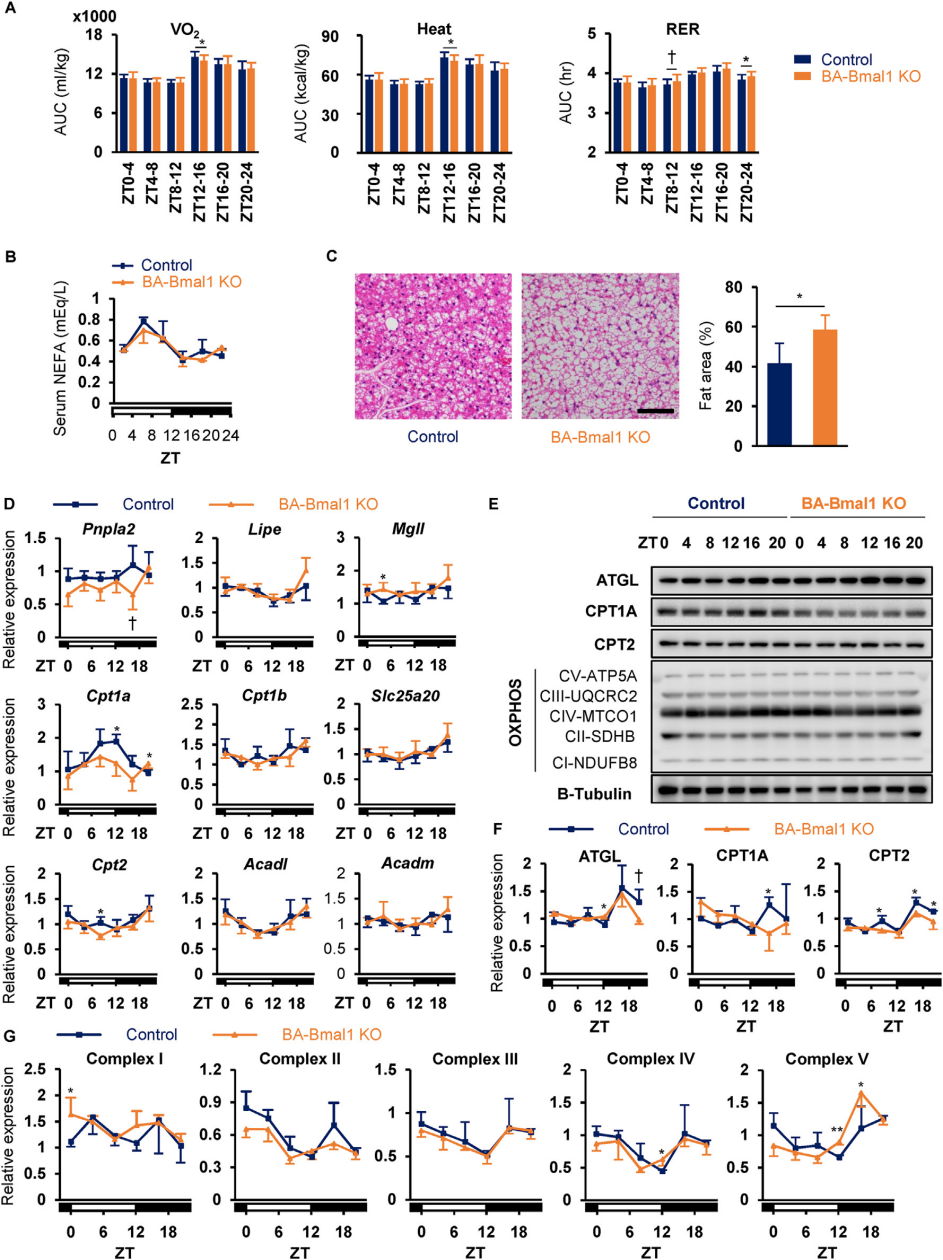
**BA-Bmal1 KO mildly impaired lipid utilization in BAT.** (A) Daily profiles of energy expenditure (VO_2_ and heat) and the respiratory exchange ratio (RER). See also Fig. S2. Data are presented as the mean +SD of 30 values obtained from five mice. †p = 0.08 and ∗p < 0.05. (B) Daily profile of serum non-esterified fatty acids (NEFA) (n = 3–4 per time point per group). (C) Representative hematoxylin and eosin staining of BAT (left) and measured fat area (right). The BAT samples were obtained at ZT 6. Scale bar indicates 100 μm. Data are presented as the mean +SD (n = 3 for control and n = 4 for BA-Bmal1 KO). ∗p < 0.05. (D–G) Daily expression profiles of fatty acid utilization-related genes in BAT. mRNA levels of fatty acid utilization-related genes (D) were analyzed by quantitative PCR. The protein expression of fatty acid utilization-related genes (F) and assembly of mitochondrial oxidative phosphorylation complexes (G) were quantified by Western blotting analyses (E). Data are presented as the mean and SD of 3–4 mice per time point per group. †p < 0.10 and ∗p < 0.05 at each time point.

Both whole-body oxygen consumption and energy expenditure were mostly comparable between genotypes but were significantly lower in the BA-Bmal1 KO mice during the early active phase (Figure 4A and Supplemental Fig. 2). Indirect calorimetry also revealed that the respiratory exchange ratio in the late active phase (ZT 20–24) was higher in the BA-Bmal1 KO mice, indicating a shift in energy source from fatty acids to glucose. Therefore, we then focused on the fatty acid utilization. As shown in Figure 4B, the concentrations of free fatty acids in serum, the major substrates for BAT thermogenesis [40], did not differ for 24 h between genotypes. However, as reported in systemic *Bmal1* KO mice [41], histological analyses showed a significant increase in the BAT adipocyte area in the BA-Bmal1 KO mice (Figure 4C). It has been shown in the liver of mice that most of the proteins related to fatty acid utilization (uptake and oxidation) and the respiratory chain have circadian patterns of expression under regulation of the intracellular clock [42]. Therefore, we further investigated the daily expression rhythms of these proteins in the BAT. As shown in Figure 4D,E, and 4F, adipose triglyceride lipase (ATGL, encoded by *Pnpla2*) tended to exhibit dampened 24 h expression rhythms of mRNA and protein in the BA-Bmal1 KO mice compared to the control mice. Moreover, carnitine palmitoyltransferase (CPT) 1A and CPT2, which are enzymes essential for long-chain fatty acid influx from cytosol to the mitochondrial matrix, were expressed at significantly lower levels in the BA-Bmal1 KO mice, particularly when these levels nearly peaked in the control mice. BA-Bmal1 KO somewhat increased the levels of proteins involved in the assembly of mitochondrial oxidative phosphorylation complexes (Figure 4E,G), suggesting minimal harmful effects of this KO on mitochondria. Collectively, these results suggest that BA-Bmal1 KO mildly impairs lipid utilization in the BAT possibly due to the disruption of daily rhythms in fatty acid metabolism.

### 3.5 BA-Bmal1 KO reduced energy charges in BAT

**Figure 5:**
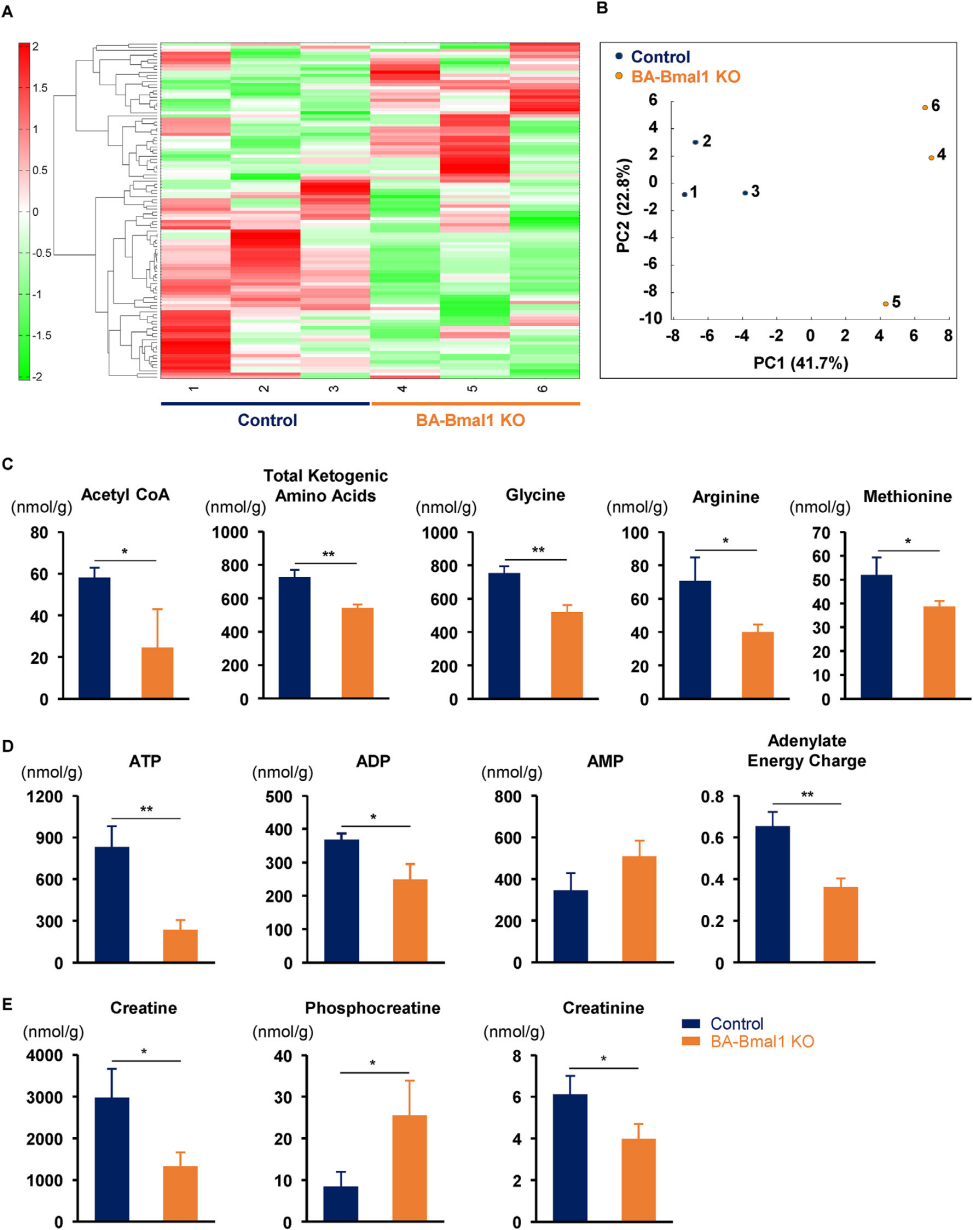
BA-Bmal1 KO reduced energy charges in BAT. (A and B) Hierarchical cluster analysis (A) and principal component analysis (B) of the metabolome data. See also Supplemental Table 2 and Supplemental Fig. 3. The BAT samples were obtained at ZT 20 from six mice (three mice in each genotype), and the metabolome analysis was conducted using CE-TOFMS and CE-MS/MS. (C–E) The levels of acetyl CoA and amino acids (C), adenylates and adenylate energy charge (D), and creatines (E) in BAT. The adenylate energy charge was calculated using the formula [43]: [ATP + 0.5 × ADP]/[ATP + ADP + AMP]. Data are presented as the mean +SD of three mice. ∗p < 0.05 and ∗∗p < 0.01.

Consistent with these results, a hierarchical cluster analysis (Figure 5A) and principal component analysis (Figure 5B) of the metabolome data (Supplemental Table 2 and Supplemental Fig. 3) showed that the BAT samples of the BA-Bmal1 KO mice were clearly distinguishable from those of the control mice. The amount of acetyl CoA, a metabolite derived from fatty acid catabolism, was significantly lower in the BA-Bmal1 KO mice than the control mice (Figure 5C). In addition, ketogenic amino acids, which can also produce acetyl CoA, and several glucogenic amino acids including glycine, arginine, and methionine, decreased in the BA-Bmal1 KO mice. Furthermore, not only ATP and ADP concentrations but the adenylate energy charge, an index of cellular energy status [43], was significantly lower in the BA-Bmal1 KO mice (Figure 5D). Notably, both creatine and creatinine also decreased in the BA-Bmal1 KO mice (Figure 5E). Creatine is known to be synthesized from glycine, arginine, and methionine, metabolize to creatinine [44], and have a key role in UCP1-independent thermogenesis in the BAT [45]. Specifically, the BAT might produce heat through the hydrolysis of phosphocreatine, although the precise mechanisms underlying the regulation of this process remain unclear [46,47]. Because phosphocreatine increased in the BA-Bmal1 KO mice, it is also possible that BA-Bmal1 KO impairs this UCP1-independent thermogenesis.

### 3.6 BA-Bmal1 KO mice were more prone to diet-induced obesity

**Figure 6:**
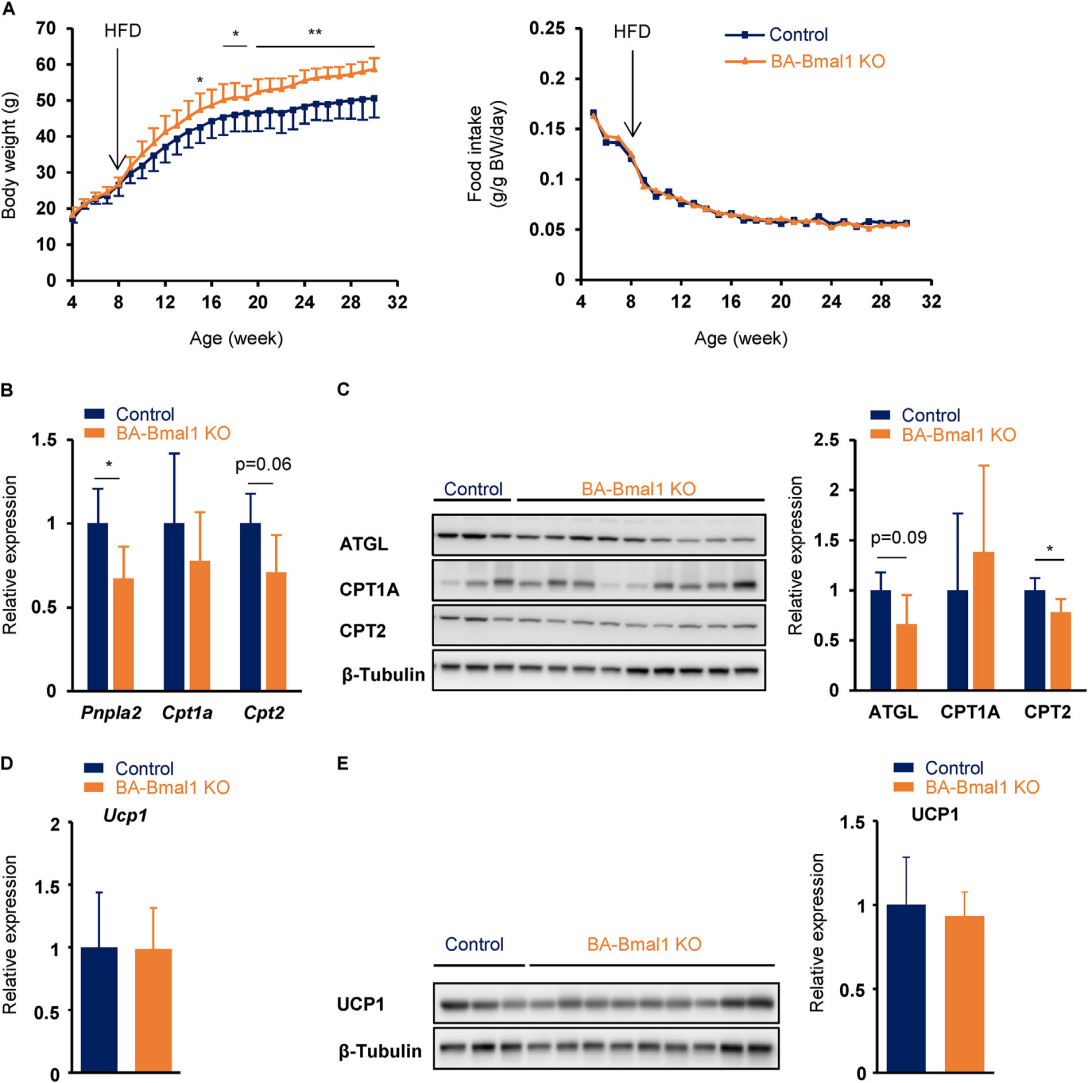
**The BA-Bmal1 KO mice were more prone to diet-induced obesity.** (A) Body weight (left) and food intake (right) during high-fat diet feeding. See also Supplemental Fig. 4. The mice were housed three animals per cage from 4 weeks of age. High-fat diet feeding started at 8 weeks of age. In the first experiment, BA-Bmal1 KO mice (n = 9) and littermate controls (n = 3) were housed mixed, and their body weights and food intake were measured weekly in a blinded fashion without determining the genotype. In the second experiment, the mice (n = 6, each genotype) were separated by their genotypes. Data are presented as the mean and SD (n = 9 for control and n = 15 for BA-Bmal1 KO) or means of two cages in the second experiment. ∗p < 0.05 and ∗∗p < 0.01 at each time point. (B–E) mRNA (B and D) and protein expression (C and E) of fatty acid utilization-related genes (B and C) and UCP1 (D and E) in BAT of the diet-induced obese mice. The samples were obtained at approximately ZT 6 from 12 mice in the first experiment. Data are presented as the mean +SD. ∗p < 0.05.

We investigated whether BA-Bmal1 KO leads to obesity. Body weight did not differ between genotypes when they were fed a normal chow diet (Figure 1D). However, after over 7 weeks of high-fat diet feeding, the BA-Bmal1 KO mice gained significantly more weight than their littermate controls with no change in food intake (Figure 6A and Supplemental Fig. 4). Consistent with body weight gain, the weights of most of the adipose tissues were higher in the BA-Bmal1 KO mice than the control mice (Table 1). Quantitative PCR (Figure 6B) and Western blotting analyses (Figure 6C) reconfirmed the effects of BA-Bmal1 KO on the expression of fatty acid utilization-related genes even under a high-fat diet. However, the differences in UCP1 expression between genotypes, which were observed in the mice fed a normal chow diet (Figure 1B,C), disappeared after high-fat diet feeding (Figure 6D,E). This may not be surprising because UCP1 expression is reported to be increased in normal mice fed a high-fat diet [48]. Rather, this disappearance could potentially contribute to the exacerbation of obesity in the BA-Bmal1 KO mice. Taken together, these results clearly indicate that disruption of the circadian clock in BAT is an exacerbating factor of obesity.

## 4 Discussion

Chang et al. [49] analyzed BA-Bmal1 KO mice harboring *Bmal1*-floxed alleles (RRID:IMSR_JAX:007668) and showed that, compared to wild-type mice, both the core body temperature and blood pressure were significantly lower during the rest phase at 22 °C with no change in locomotor activity. However, the BA-Bmal1 KO mice used in this study, which harbored RRID:MGI:5613396, exhibited a similar core body temperature rhythm with increases in both locomotor activity and shivering compared to the control mice. We also found that the mice with lower body temperatures were more active, suggesting that behavioral thermogenesis, that is, body heat produced by increased locomotor activity [38], increased in the BA-Bmal1 KO mice. The decrease in SNS activity also indicated compensatory thermogenesis in the skeletal muscle, which is not mediated by the sympathetic but somatic nervous system [38,39]. Although the reason for those discrepancies between the phenotypes of the two BA-Bmal1 KO strains remains unclear, a significant phenotypical difference between liver-specific *Bmal1* KO mice generated using IMSR_JAX:007668 and those with MGI:5613396 has also been reported. Specifically, hepatic triglyceride accumulation after high-fat diet feeding was observed in the former [50] but not in the latter mice [25]. Interestingly, this phenotype was also observed in systemic *Bmal1* KO mice using MGI:5613396 and *Pgk*-Cre mice [25]. Thus, the two *Bmal1*-floxed alleles appear to possess somewhat different characteristics. Our results confirmed the possibility that BA-Bmal1 KO impairs non-shivering thermogenesis in BAT. Chang et al. [49] concluded that decreases in the blood pressure and heart rate in BA-Bmal1 KO mice might be due to impairment of local angiotensin II production in the perivascular adipose tissue. However, our data raise a possibility that decreases in the blood pressure and heart rate may occur, at least in part, as a consequence of reduced SNS activation.

One of the possible mechanisms underlying the link between circadian clocks and obesity is the regulation of adipose functions by the intracellular clock in white adipocytes [17,18]. Reportedly, adipocyte-specific *Bmal1* KO mice generated using *aP2*-Cre mice have decreased concentrations of polyunsaturated fatty acids in both the plasma and hypothalamus, which regulates food intake, and consequently have increased food intake and gain more body weight and fat mass compared to control mice, particularly when fed a high-fat diet [51]. These effects of *Bmal1* deletion were considered due to the reduced expression of *Elovl6* and *Scd1*, the direct clock-controlled genes involved in fatty acid biosynthesis in white adipose tissue. However, because *Bmal1* was knocked out not only in the white adipose tissue but also in the BAT of this strain, it is possible that the weight-increasing effects of adipocyte-specific *Bmal1* KO were caused in part by the impairment of BAT thermogenesis.

Proteomics analyses have shown that most of the enzymes involved in fatty acid uptake and oxidation exhibit 24 h expression rhythmicity in the mitochondria of mouse liver [42]. In particular, the rhythm of CPT1A disappeared in *Per1/2*-deficient mice, suggesting that the daily rhythm of fatty acid utilization is controlled by the circadian clock. This study confirmed these findings in BAT and further indicated the pathophysiological impact of this disrupted rhythmicity. The fact that the overall 24 h BAT function was not obviously impaired in the BA-Bmal1 KO mice may highlight the importance of circadian rhythms in controlling energy homeostasis.

Recent studies have suggested that futile creatine cycling is involved in UCP1-independent thermogenesis of BAT [45]. Genetic depletion of creatine levels in adipocytes through either synthesis [52] or uptake from the circulation [53] impairs thermogenesis and potentiates diet-induced obesity. In this study, BA-Bmal1 KO decreased BAT creatine levels but increased phosphocreatine levels, suggesting the impairment of futile creatine cycling. Further studies are needed to elucidate the detailed mechanism underlying the association between creatine cycling and the intracellular circadian clock.

In conclusion, BA-Bmal1 KO disrupted the 24 h rhythmicity of fatty acid utilization in BAT and reduced BAT thermogenesis despite the increased expression of UCP1. Because this impairment was compensated by other types of thermogenesis, that is, shivering and behavioral thermogenesis, the phenotypic effects were minimal under normal conditions. However, the impact of BA-Bmal1 KO on the development of obesity became obvious when the mice were fed a high-fat diet. These results provide novel insights into the mechanisms underlying the association between impaired circadian clocks and obesity.

## Funding

This work study supported by 10.13039/501100001691JSPS KAKENHI grant numbers JP18K08470, JP19H01617, JP19K09776, JP20H03996, and JP20K21651, 10.13039/100009619AMED Wise and Kanazawa University CHOZEN Project.

## Author contributions

N.H. designed the study, conducted the experiments, analyzed the data, and wrote the manuscript. N.N., J.M., M.T.I, Z.J, and K.H. performed the experiments. M.M., M.O., H·F., T.D., T.F., Y.M., T.O., S·S., S·K., and A.F. provided resources and expertise. H.A. conceptualized the study, designed the research, conducted the experiments, analyzed the data, and co-wrote the manuscript with input from all of the co-authors.

## Acknowledgments

We are grateful to Tomoharu Yasuda, Kunikazu Saikawa, Kenta Takahashi, and Yuko Tsurumi for technical assistance. We also thank Prof. Hiroshi Kawasaki for his support.

## Declaration of competing interest

The authors have no conflicts of interest to declare.
