# Supplementary material for: Brown adipocyte-specific knockout of Bmal1 causes mild but significant thermogenesis impairment in mice
Source: Mol Metab. 2021 Mar 3;49:101202. doi: 10.1016/j.molmet.2021.101202 (PMC8042177; doi:10.1016/j.molmet.2021.101202)
Supplement: Supplementary file 1 — Multimedia component 1 [file mmc1.docx]

Supplemental Table 1. TaqMan gene expression assays used in this study.

| Gene | NCBI Reference Sequence | Assay ID | Target exons |
| --- | --- | --- | --- |
| *Bmal1* | NM_007489.4 | Mm00500222_m1 | 7–8 |
| *Per1* | NM_011065.4 | Mm00501813_m1 | 18–19 |
| *Cry1* | NM_007771.3 | Mm00514392_m1 | 1–2 |
| *Nr1d1* | NM_145434.4 | Mm00520708_m1 | 1–2 |
| *Adrb3* | NM_013462.3 | Mm02601819_g1 | 3–4 |
| *Ucp1* | NM_009463.3 | Mm01244861_m1 | 5–6 |
| *Ppargc1a* | NM_008904.2 | Mm01208835_m1 | 7–8 |
| *Pnpla2* | NM_025802.3 | Mm00503040_m1 | 1–2 |
| *Lipe* | NM_010719.5 | Mm00495359_m1 | 8–9 |
| *Mgll* | NM_011844.4 | Mm00449274_m1 | 2–3 |
| *Cpt1a* | NM_013495.2 | Mm01231183_m1 | 13–14 |
| *Cpt1b* | NM_009948.2 | Mm00487200_m1 | 17–18 |
| *Slc25a20* | NM_020520.4 | Mm00451571_m1 | 4–5 |
| *Cpt2* | NM_009949.2 | Mm00487205_m1 | 4–5 |
| *Acadl* | NM_007381.4 | Mm00599660_m1 | 8–9 |
| *Acadm* | NM_007382.5 | Mm01323360_g1 | 3–4 |
| *Rplp0* | NM_007475.5 | Mm00725448_s1 | 7 |
