# Supplementary material for: Brown adipocyte-specific knockout of Bmal1 causes mild but significant thermogenesis impairment in mice
Source: Mol Metab. 2021 Mar 3;49:101202. doi: 10.1016/j.molmet.2021.101202 (PMC8042177; doi:10.1016/j.molmet.2021.101202)
Supplement: Supplementary file 2 — Multimedia component 2 [file mmc2.pdf]

**Supplemental Table 2.** Absolute concentrations of 116 metabolites.

| | HMT DB | | | | Concentration (nmol/g) | | | | | | | | | | *t*-test |
|---|---|---|---|---|---|---|---|---|---|---|---|---|---|---|---|
| ID | Compound name | Pathway Label | PubChem CID | HMDB ID | Control | | | KO | | | Control | | KO | | KO vs Control |
| | | | | | 1 | 2 | 3 | 4 | 5 | 6 | Mean | S.D. | Mean | S.D. | P-value |
| A_0001 | $NAD^{+}$ | NAD+ | 5893 | HMDB0000902 | 574 | 480 | 556 | 332 | 471 | 394 | 536 | 50 | 399 | 70 | 0.05 |
| A_0002 | cAMP | cAMP | 6076 | HMDB0000058 | 1.3 | 1.0 | 1.4 | 0.9 | 1.2 | 1.1 | 1.2 | 0.2 | 1.1 | 0.2 | 0.33 |
| A_0003 | cGMP | cGMP | 24316 | HMDB0001314 | N.D. | 0.02 | N.D. | N.D. | N.D. | N.D. | 0.02 | N.A. | N.A. | N.A. | N.A. |
| A_0004 | NADH | NADH | 439153 | HMDB0001487 | 6.0 | 5.6 | 6.5 | 6.0 | 4.9 | 6.8 | 6.0 | 0.5 | 5.9 | 1.0 | 0.82 |
| A_0005 | Xanthine | Xanthine | 1188 | HMDB0000292 | 3.9 | 5.9 | 4.3 | 6.8 | 6.3 | 9.3 | 4.7 | 1.1 | 7.5 | 1.6 | 0.07 |
| A_0006 | ADP-ribose | ADP-Rib | 445794 | HMDB0001178 | 114 | 107 | 126 | 145 | 146 | 148 | 116 | 9.7 | 146 | 1.6 | <0.01 |
| A_0007 | Mevalonic acid | Mevalonic acid | 134965 | HMDB0000227 | N.D. | N.D. | N.D. | N.D. | N.D. | N.D. | N.A. | N.A. | N.A. | N.A. | N.A. |
| A_0008 | UDP-glucose | UDP-Glc | 8629 | HMDB0000286 | 93 | 79 | 71 | 42 | 91 | 25 | 81 | 11 | 53 | 35 | 0.24 |
| A_0009 | Uric acid | Uric acid | 1175 | HMDB0000289 | 12 | 14 | 12 | 12 | 17 | 15 | 13 | 1.4 | 15 | 2.3 | 0.25 |
| A_0010 | $NADP^{+}$ | NADP+ | 5886 | HMDB0000217 | 56 | 46 | 45 | 41 | 53 | 46 | 49 | 6.2 | 47 | 6.3 | 0.67 |
| A_0011 | IMP | IMP | 8582 | HMDB0000175 | 118 | 170 | 149 | 225 | 169 | 240 | 146 | 26 | 211 | 37 | 0.07 |
| A_0012 | Sedoheptulose 7-phosphate | S7P | 165007 | HMDB0001068 | 4.9 | N.D. | 11 | 2.7 | 26 | N.D. | 7.8 | 4.2 | 14 | 17 | 0.65 |
| A_0013 | Glucose 6-phosphate | G6P | 5958 | HMDB0001401 | 239 | 263 | 358 | 258 | 277 | 229 | 287 | 63 | 255 | 24 | 0.46 |
| A_0014 | Fructose 6-phosphate | F6P | 603 | HMDB0000124 | 76 | 83 | 113 | 79 | 84 | 79 | 91 | 20 | 81 | 2.6 | 0.48 |
| A_0015 | Fructose 1-phosphate | D-F1P | 439394 | HMDB0001076 | 28 | 11 | 18 | 17 | 35 | 9.5 | 19 | 9.0 | 21 | 13 | 0.89 |
| A_0016 | Galactose 1-phosphate | Gal1P | 123912 | HMDB0000645 | 4.3 | 3.0 | 4.3 | 4.9 | 6.0 | 3.2 | 3.9 | 0.7 | 4.7 | 1.4 | 0.42 |
| A_0017 | Glucose 1-phosphate | G1P | 65533 | HMDB0001586 | 16 | 17 | 19 | 14 | 16 | 15 | 17 | 1.9 | 15 | 1.0 | <0.05 |
| A_0018 | Acetoacetyl CoA | AAcCoA | 92153 | HMDB0001484 | N.D. | N.D. | N.D. | N.D. | N.D. | N.D. | N.A. | N.A. | N.A. | N.A. | N.A. |
| A_0019 | Acetyl CoA | AcCoA | 444493 | HMDB0001206 | 57 | 63 | 54 | 11 | 46 | 17 | 58 | 4.8 | 25 | 18 | <0.05 |
| A_0020 | Folic acid | Folic acid | 6037 | HMDB0000121 | N.D. | N.D. | N.D. | 0.2 | N.D. | N.D. | N.A. | N.A. | 0.2 | N.A. | N.A. |
| A_0021 | Ribose 5-phosphate | R5P | 439167 | HMDB0001548 | 12 | 8.8 | 11 | 15 | 17 | 12 | 10 | 1.5 | 15 | 2.5 | 0.07 |
| A_0022 | CoA | CoA | 87642 | HMDB0001423 | 101 | 89 | 91 | 112 | 96 | 134 | 93 | 6.4 | 114 | 19 | 0.15 |
| A_0023 | Ribose 1-phosphate | R1P | 439236 | HMDB0001489 | 13 | 13 | 9.1 | 14 | 15 | 16 | 12 | 2.5 | 15 | 1.4 | 0.16 |
| A_0024 | Ribulose 5-phosphate | Ru5P | 439184 | HMDB0000618 | 5.9 | 3.8 | 6.1 | 5.3 | 7.7 | 6.2 | 5.3 | 1.3 | 6.4 | 1.2 | 0.34 |
| A_0025 | Xylulose 5-phosphate | X5P | 439190 | HMDB0000868 | N.D. | N.D. | N.D. | N.D. | 3.3 | N.D. | N.A. | N.A. | 3.3 | N.A. | N.A. |
| A_0026 | Erythrose 4-phosphate | E4P | 122357 | HMDB0001321 | N.D. | N.D. | N.D. | N.D. | N.D. | N.D. | N.A. | N.A. | N.A. | N.A. | N.A. |
| A_0027 | HMG CoA | HMG-CoA | 445127 | HMDB0001375 | 0.3 | 0.4 | 0.5 | 0.3 | 0.4 | N.D. | 0.4 | 0.07 | 0.3 | 0.04 | 0.24 |
| A_0028 | Glyceraldehyde 3-phosphate | Glyceraldehyde 3-phosphate | 729 | HMDB0001112 | 7.1 | 9.9 | 10 | 21 | 20 | 17 | 9.1 | 1.8 | 19 | 2.2 | <0.01 |
| A_0029 | NADPH | NADPH | 5884 | HMDB0000221 | 14 | 14 | 7.4 | 10 | 8.9 | 6.0 | 12 | 3.8 | 8.3 | 2.1 | 0.24 |
| A_0030 | Malonyl CoA | Malonyl-CoA | 644066 | HMDB0001175 | 0.4 | 0.5 | 0.5 | 0.4 | 0.3 | 0.5 | 0.4 | 0.03 | 0.4 | 0.10 | 0.26 |
| A_0031 | Phosphocreatine | Phosphocreatine | 9548602 | HMDB0001511 | 10 | 4.3 | 11 | 34 | 18 | 24 | 8.4 | 3.6 | 26 | 8.3 | <0.05 |
| A_0032 | XMP | XMP | 73323 | HMDB0001554 | 0.2 | 0.13 | 0.15 | 0.2 | 0.2 | 0.2 | 0.14 | 0.012 | 0.2 | 0.02 | <0.05 |
| A_0033 | Dihydroxyacetone phosphate | DHAP | 668 | HMDB0001473 | 255 | 284 | 351 | 298 | 459 | 310 | 297 | 49 | 356 | 90 | 0.37 |
| A_0034 | Adenylosuccinic acid | Succinyl AMP | 447145 | HMDB0000536 | 22 | 20 | 15 | 38 | 29 | 27 | 19 | 3.7 | 32 | 6.1 | <0.05 |
| A_0035 | Fructose 1,6-diphosphate | F1,6P | 172313 | HMDB0001058 | 178 | 254 | 234 | 279 | 351 | 222 | 222 | 39 | 284 | 64 | 0.23 |
| A_0036 | 6-Phosphogluconic acid | 6-PG | 91493 | HMDB0001316 | 55 | 27 | 47 | 77 | 82 | 40 | 43 | 14 | 66 | 23 | 0.21 |
| A_0037 | *N*-Carbamoylaspartic acid | Carbamoyl-Asp | 93072 | HMDB0000828 | 1.6 | 0.6 | 0.3 | 0.4 | 0.4 | 0.11 | 0.8 | 0.7 | 0.3 | 0.2 | 0.28 |
| A_0038 | PRPP | PRPP | 7339 | HMDB0000280 | 20 | 7.6 | 10 | 7.5 | 5.2 | 2.2 | 13 | 6.8 | 5.0 | 2.6 | 0.14 |
| A_0039 | 2-Phosphoglyceric acid | 2-PG | 439278 | HMDB0003391 | 1.0 | 1.8 | 0.7 | 0.5 | 0.8 | 0.5 | 1.2 | 0.6 | 0.6 | 0.2 | 0.20 |
| A_0040 | 2,3-Diphosphoglyceric acid | Diphosphoglycerate | 186004 | HMDB0001294 | 169 | 121 | 90 | 89 | 131 | 117 | 127 | 40 | 112 | 21 | 0.61 |
| A_0041 | 3-Phosphoglyceric acid | 3-PG | 439183 | HMDB0000807 | 9.2 | 15 | 6.4 | 2.2 | 4.8 | 3.1 | 10 | 4.6 | 3.4 | 1.3 | 0.06 |
| A_0042 | Phosphoenolpyruvic acid | PEP | 1005 | HMDB0000263 | 0.8 | 2.4 | N.D. | N.D. | N.D. | N.D. | 1.6 | 1.2 | N.A. | N.A. | N.A. |
| A_0043 | GMP | GMP | 6804 | HMDB0001397 | 123 | 111 | 101 | 133 | 109 | 124 | 112 | 11 | 122 | 12 | 0.33 |
| A_0044 | AMP | AMP | 6083 | HMDB0000045 | 306 | 292 | 441 | 419 | 560 | 543 | 346 | 82 | 507 | 77 | 0.07 |
| A_0045 | 2-Oxoisovaleric acid | 2-KIV | 49 | HMDB0000019 | N.D. | N.D. | 7.3 | N.D. | 6.8 | N.D. | 7.3 | N.A. | 6.8 | N.A. | N.A. |
| A_0046 | GDP | GDP | 8977 | HMDB0001201 | 43 | 31 | 49 | 47 | 51 | 42 | 41 | 8.8 | 47 | 4.9 | 0.39 |
| A_0047 | Lactic acid | Lactic acid | 612 | HMDB0000190 | 9,545 | 9,420 | 11,069 | 7,189 | 9,996 | 6,607 | 10,011 | 919 | 7,931 | 1,812 | 0.15 |
| A_0048 | ADP | ADP | 6022 | HMDB0001341 | 355 | 388 | 363 | 203 | 298 | 244 | 369 | 17 | 248 | 47 | <0.05 |
| A_0049 | GTP | GTP | 6830 | HMDB0001273 | 123 | 95 | 110 | 54 | 67 | 43 | 110 | 14 | 55 | 12 | <0.01 |
| A_0050 | Glyoxylic acid | Glyoxylic acid | 760 | HMDB0000119 | N.D. | N.D. | N.D. | N.D. | N.D. | N.D. | N.A. | N.A. | N.A. | N.A. | N.A. |
| A_0051 | ATP | ATP | 5957 | HMDB0000538 | 843 | 973 | 671 | 204 | 317 | 185 | 829 | 152 | 235 | 71 | <0.01 |
| A_0052 | Glycerol 3-phosphate | Glycerol 3-phosphate | 439162 | HMDB0000126 | 894 | 648 | 468 | 294 | 662 | 258 | 670 | 214 | 405 | 224 | 0.21 |
| A_0053 | Glycolic acid | Glycolic acid | 757 | HMDB0000115 | 2.1 | N.D. | N.D. | N.D. | N.D. | N.D. | 2.1 | N.A. | N.A. | N.A. | N.A. |
| A_0054 | Pyruvic acid | Pyruvic acid | 1060 | HMDB0000243 | 175 | 174 | 199 | 163 | 172 | 172 | 183 | 14 | 169 | 5.3 | 0.20 |
| A_0055 | *N*-Acetylglutamic acid | N-AcGlu | 70914 | HMDB0001138 | 26 | 14 | 31 | 11 | 17 | 19 | 24 | 8.5 | 16 | 4.0 | 0.23 |
| A_0056 | 2-Hydroxyglutaric acid | 2-Hydroxyglutaric acid | 43 | HMDB0000606 | 22 | 12 | 6.8 | 19 | 20 | 10 | 13 | 7.5 | 16 | 5.5 | 0.60 |
| A_0057 | Carbamoylphosphate | Carbamoyl-P | 278 | HMDB0001096 | N.D. | N.D. | N.D. | N.D. | N.D. | N.D. | N.A. | N.A. | N.A. | N.A. | N.A. |
| A_0058 | Succinic acid | Succinic acid | 1110 | HMDB0000254 | 1,031 | 772 | 788 | 735 | 1,158 | 644 | 863 | 145 | 845 | 274 | 0.93 |
| A_0059 | Malic acid | Malic acid | 525 | HMDB0000156 | 958 | 623 | 620 | 993 | 1,032 | 512 | 734 | 194 | 846 | 290 | 0.61 |
| A_0060 | 2-Oxoglutaric acid | 2-OG | 51 | HMDB0000208 | 0.5 | N.D. | N.D. | N.D. | 5.4 | N.D. | 0.5 | N.A. | 5.4 | N.A. | N.A. |
| A_0061 | Fumaric acid | Fumaric acid | 444972 | HMDB0000134 | 412 | 286 | 275 | 411 | 439 | 228 | 324 | 76 | 359 | 115 | 0.68 |
| A_0062 | Citric acid | Citric acid | 311 | HMDB0000094 | 623 | 606 | 651 | 748 | 858 | 569 | 627 | 23 | 725 | 146 | 0.31 |
| A_0063 | *cis*-Aconitic acid | cis-Aconitic acid | 643757 | HMDB0000072 | 9.8 | 9.9 | 14 | 16 | 19 | 9.7 | 11 | 2.6 | 15 | 4.8 | 0.31 |
| A_0064 | Isocitric acid | Isocitric acid | 1198 | HMDB0000193 | 0.7 | N.D. | N.D. | 0.7 | 3.1 | N.D. | 0.7 | N.A. | 1.9 | 1.7 | N.A. |
| C_0001 | Urea | Urea | 1176 | HMDB0000294 | 5,215 | 4,249 | 3,791 | 4,100 | 4,155 | 3,731 | 4,418 | 727 | 3,995 | 231 | 0.39 |
| C_0002 | Gly | Gly | 750 | HMDB0000123 | 728 | 802 | 729 | 473 | 557 | 529 | 753 | 43 | 520 | 43 | <0.01 |
| C_0003 | Putrescine | Putrescine | 1045 | HMDB0001414 | 5.1 | 4.0 | 5.1 | 3.5 | 5.1 | 3.8 | 4.8 | 0.7 | 4.1 | 0.8 | 0.38 |
| C_0004 | Ala | Ala | 602 | HMDB0000161 | 1,797 | 1,082 | 1,050 | 1,581 | 918 | 1,480 | 1,310 | 422 | 1,326 | 357 | 0.96 |
| C_0005 | Sarcosine | Sarcosine | 1088 | HMDB0000271 | 9.8 | 7.3 | 6.7 | 5.6 | 5.1 | 8.9 | 8.0 | 1.6 | 6.5 | 2.1 | 0.40 |
| C_0006 | β-Ala | b-Ala | 239 | HMDB0000056 | 18 | 13 | 11 | 9.8 | 12 | 9.5 | 14 | 3.5 | 10 | 1.3 | 0.17 |
| C_0007 | *N*,*N*-Dimethylglycine | DMG | 673 | HMDB0000092 | 5.3 | 4.1 | 3.9 | 3.4 | 4.4 | 3.9 | 4.4 | 0.7 | 3.9 | 0.5 | 0.34 |
| C_0008 | γ-Aminobutyric acid | g-Aminobutyric acid | 119 | HMDB0000112 | 2.4 | 2.9 | 2.6 | 2.5 | 2.2 | 3.3 | 2.6 | 0.3 | 2.7 | 0.6 | 0.92 |
| C_0009 | Choline | Choline | 305 | HMDB0000097 | 15 | 40 | 12 | 3.8 | 23 | 6.1 | 22 | 15 | 11 | 10 | 0.34 |
| C_0010 | Ser | Ser | 617 | HMDB0000187 | 268 | 257 | 259 | 192 | 205 | 205 | 261 | 5.7 | 201 | 7.4 | <0.01 |
| C_0011 | Carnosine | Carnosine | 439224 | HMDB0000033 | 13 | 24 | 13 | N.D. | 5.6 | N.D. | 17 | 6.4 | 5.6 | N.A. | <0.05 |
| C_0012 | Creatinine | Creatinine | 588 | HMDB0000562 | 5.8 | 7.1 | 5.4 | 3.4 | 4.8 | 3.7 | 6.1 | 0.9 | 4.0 | 0.7 | <0.05 |
| C_0013 | Pro | Pro | 614 | HMDB0000162 | 182 | 139 | 131 | 205 | 127 | 178 | 151 | 27 | 170 | 39 | 0.53 |
| C_0014 | Val | Val | 1182 | HMDB0000883 | 131 | 117 | 116 | 114 | 93 | 108 | 122 | 8.5 | 105 | 11 | 0.11 |
| C_0015 | Betaine | Betaine | 247 | HMDB0000043 | 252 | 141 | 188 | 138 | 179 | 127 | 194 | 56 | 148 | 27 | 0.27 |
| C_0016 | Thr | Thr | 6288 | HMDB0000167 | 380 | 304 | 309 | 258 | 234 | 257 | 331 | 43 | 250 | 14 | <0.05 |
| C_0017 | Homoserine | Homoserine | 12647 | HMDB0000719 | 1.8 | 1.4 | 1.1 | 0.8 | 1.4 | N.D. | 1.4 | 0.4 | 1.1 | 0.4 | 0.19 |
| C_0018 | Betaine aldehyde | BTL | 249 | HMDB0001252 | N.D. | 0.03 | N.D. | N.D. | N.D. | N.D. | 0.03 | N.A. | N.A. | N.A. | N.A. |
| C_0019 | Cys | Cys | 594 | HMDB0000574 | 2.0 | 1.9 | N.D. | 2.7 | N.D. | N.D. | 2.0 | 0.07 | 2.7 | N.A. | N.A. |
| C_0020 | Hydroxyproline | Hydroxyproline | 5810 | HMDB0000725 | 44 | 44 | 37 | 29 | 30 | 27 | 42 | 4.3 | 29 | 1.4 | <0.01 |
| C_0021 | Creatine | Creatine | 586 | HMDB0000064 | 2,527 | 3,766 | 2,629 | 1,136 | 1,721 | 1,127 | 2,974 | 688 | 1,328 | 340 | <0.05 |
| C_0022 | Leu | Leu | 857 | HMDB0000687 | 87 | 87 | 94 | 90 | 69 | 78 | 90 | 3.9 | 79 | 10 | 0.18 |
| C_0023 | Ile | Ile | 791 | HMDB0000172 | 43 | 45 | 45 | 41 | 34 | 37 | 44 | 1.2 | 37 | 3.4 | 0.03 |
| C_0024 | Asn | Asn | 236 | HMDB0000168 | 38 | 39 | 39 | 27 | 23 | 31 | 39 | 0.9 | 27 | 3.7 | <0.01 |
| C_0025 | Ornithine | Ornithine | 389 | HMDB0000214 | 23 | 25 | 23 | 20 | 18 | 21 | 24 | 1.1 | 20 | 1.6 | <0.05 |
| C_0026 | Asp | Asp | 424 | HMDB0000191 | 339 | 241 | 259 | 332 | 156 | 265 | 280 | 52 | 251 | 89 | 0.65 |
| C_0027 | Homocysteine | Homocysteine | 778 | HMDB0000742 | N.D. | N.D. | N.D. | N.D. | N.D. | N.D. | N.A. | N.A. | N.A. | N.A. | N.A. |
| C_0028 | Adenine | Adenine | 190 | HMDB0000034 | 1.2 | 1.5 | 1.6 | 0.8 | 1.4 | 1.2 | 1.5 | 0.2 | 1.2 | 0.3 | 0.23 |
| C_0029 | Hypoxanthine | Hypoxanthine | 790 | HMDB0000157 | 3.4 | 5.2 | 3.5 | 6.6 | 5.6 | 9.3 | 4.1 | 1.0 | 7.2 | 1.9 | 0.07 |
| C_0030 | Spermidine | Spermidine | 1102 | HMDB0001257 | 32 | 23 | 25 | 38 | 44 | 27 | 26 | 4.6 | 36 | 8.2 | 0.15 |
| C_0031 | Gln | Gln | 738 | HMDB0000641 | 1,235 | 1,249 | 1,271 | 824 | 1,156 | 754 | 1,252 | 18 | 912 | 215 | 0.11 |
| C_0032 | Lys | Lys | 866 | HMDB0000182 | 132 | 174 | 134 | 71 | 91 | 83 | 147 | 24 | 82 | 9.9 | <0.05 |
| C_0033 | Glu | Glu | 611 | HMDB0000148 | 885 | 601 | 439 | 553 | 346 | 683 | 642 | 226 | 527 | 170 | 0.52 |
| C_0034 | Met | Met | 876 | HMDB0000696 | 59 | 52 | 45 | 40 | 36 | 40 | 52 | 7.2 | 39 | 2.2 | <0.05 |
| C_0035 | Guanine | Guanine | 764 | HMDB0000132 | N.D. | N.D. | N.D. | N.D. | N.D. | N.D. | N.A. | N.A. | N.A. | N.A. | N.A. |
| C_0036 | His | His | 773 | HMDB0000177 | 41 | 50 | 36 | 22 | 26 | 23 | 42 | 6.6 | 24 | 2.3 | <0.05 |
| C_0037 | Carnitine | Carnitine | 85 | HMDB0000062 | 788 | 475 | 514 | 672 | 580 | 773 | 592 | 171 | 675 | 96 | 0.50 |
| C_0038 | Phe | Phe | 994 | HMDB0000159 | 47 | 43 | 43 | 33 | 36 | 33 | 44 | 2.6 | 34 | 1.7 | <0.01 |
| C_0039 | Arg | Arg | 6322 | HMDB0000517 | 63 | 87 | 62 | 36 | 45 | 39 | 71 | 14 | 40 | 4.6 | <0.05 |
| C_0040 | Citrulline | Citrulline | 9750 | HMDB0000904 | 76 | 73 | 70 | 49 | 61 | 56 | 73 | 3.1 | 56 | 6.2 | <0.05 |
| C_0041 | Tyr | Tyr | 1153 | HMDB0000158 | 59 | 44 | 49 | 43 | 39 | 53 | 51 | 7.3 | 45 | 7.1 | 0.37 |
| C_0042 | *S*-Adenosylhomocysteine | SAH | 439155 | HMDB0000939 | 1.8 | 1.4 | 1.3 | 1.3 | 0.9 | 1.4 | 1.5 | 0.3 | 1.2 | 0.3 | 0.26 |
| C_0043 | Spermine | Spermine | 1103 | HMDB0001256 | 4.2 | 3.4 | 2.6 | 3.1 | 4.6 | 3.2 | 3.4 | 0.8 | 3.6 | 0.8 | 0.70 |
| C_0044 | Trp | Trp | 1148 | HMDB0000929 | 27 | 18 | 20 | 14 | 17 | 16 | 22 | 4.3 | 16 | 1.2 | 0.08 |
| C_0045 | Cystathionine | Cystathionine | 834 | HMDB0000099 | N.D. | N.D. | N.D. | N.D. | N.D. | N.D. | N.A. | N.A. | N.A. | N.A. | N.A. |
| C_0046 | Adenosine | Adenosine | 60961 | HMDB0000050 | 16 | 12 | 16 | 12 | 17 | 19 | 15 | 2.5 | 16 | 3.7 | 0.56 |

**Supplemental Table 2.** (continued)

| ID | HMT DB | | | | Concentration (nmol/g) | | | | | | | | | | *t*-test |
|---|---|---|---|---|---|---|---|---|---|---|---|---|---|---|---|
| | Compound name | Pathway Label | PubChem CID | HMDB ID | Control | | | KO | | | Control | | KO | | KO vs |
| | | | | | 1 | 2 | 3 | 4 | 5 | 6 | Mean | S.D. | Mean | S.D. | P-value |
| C_0047 | Inosine | Inosine | 6021 | HMDB0000195 | 15 | 16 | 15 | 19 | 18 | 26 | 15 | 1.1 | 21 | 4.8 | 0.17 |
| C_0048 | Guanosine | Guanosine | 6802 | HMDB0000133 | 5.0 | 2.9 | 3.6 | 4.1 | 3.9 | 4.6 | 3.8 | 1.1 | 4.2 | 0.3 | 0.59 |
| C_0049 | Argininosuccinic acid | ArgSuccinate | 16950 | HMDB0000052 | 4.5 | 3.7 | 3.9 | 3.9 | 3.7 | 3.1 | 4.0 | 0.4 | 3.6 | 0.4 | 0.25 |
| C_0050 | Glutathione (GSSG) | GSSG | 65359 | HMDB0003337 | 212 | 129 | 153 | 86 | 189 | 108 | 165 | 43 | 128 | 54 | 0.40 |
| C_0051 | Glutathione (GSH) | GSH | 124886 | HMDB0000125 | 470 | 383 | 438 | 515 | 477 | 560 | 431 | 44 | 517 | 42 | 0.07 |
| C_0052 | *S*-Adenosylmethionine | SAM | 34755 | HMDB0001185 | 18 | 14 | 14 | 12 | 12 | 10 | 16 | 2.0 | 11 | 0.8 | <0.05 |
| - | Adenylate Energy Charge | No Label | | | 0.7 | 0.7 | 0.6 | 0.4 | 0.4 | 0.3 | 0.7 | 0.07 | 0.4 | 0.04 | <0.01 |
| - | Total Adenylate | No Label | | | 1,504 | 1,653 | 1,475 | 826 | 1,174 | 972 | 1,544 | 96 | 991 | 175 | <0.01 |
| - | Guanylate Energy Charge | No Label | | | 0.5 | 0.5 | 0.5 | 0.3 | 0.4 | 0.3 | 0.5 | 0.03 | 0.3 | 0.05 | <0.05 |
| - | Total Guanylate | No Label | | | 289 | 238 | 261 | 235 | 227 | 208 | 262 | 25 | 223 | 14 | 0.08 |
| - | GSH/GSSG | No Label | | | 2.2 | 3.0 | 2.9 | 6.0 | 2.5 | 5.2 | 2.7 | 0.4 | 4.6 | 1.8 | 0.15 |
| - | Total Glutathione | No Label | | | 894 | 642 | 744 | 686 | 854 | 777 | 760 | 127 | 772 | 84 | 0.90 |
| - | NADPH/NADP+ | No Label | | | 0.2 | 0.3 | 0.2 | 0.2 | 0.2 | 0.13 | 0.2 | 0.07 | 0.2 | 0.06 | 0.35 |
| - | NADH/NAD+ | No Label | | | 0.010 | 0.012 | 0.012 | 0.02 | 0.010 | 0.02 | 0.011 | 7.3E-04 | 0.02 | 0.004 | 0.25 |
| - | Lactate/Pyruvate | No Label | | | 54 | 54 | 56 | 44 | 58 | 38 | 55 | 0.7 | 47 | 10 | 0.25 |
| - | Glycerol 3-phosphate/DHAP | No Label | | | 3.5 | 2.3 | 1.3 | 1.0 | 1.4 | 0.8 | 2.4 | 1.1 | 1.1 | 0.3 | 0.12 |
| - | Total Amino Acids | No Label | | | 6,543 | 5,434 | 5,132 | 4,950 | 4,208 | 4,894 | 5,703 | 743 | 4,684 | 413 | 0.11 |
| - | Total Essential Amino Acids | No Label | | | 948 | 890 | 843 | 683 | 636 | 677 | 894 | 53 | 665 | 25 | <0.01 |
| - | Total Non-essential Amino Acids | No Label | | | 5,596 | 4,544 | 4,290 | 4,267 | 3,572 | 4,217 | 4,810 | 692 | 4,019 | 388 | 0.16 |
| - | Total Glucogenic Amino Acids | No Label | | | 6,324 | 5,173 | 4,904 | 4,789 | 4,048 | 4,732 | 5,467 | 754 | 4,523 | 412 | 0.13 |
| - | Total Ketogenic Amino Acids | No Label | | | 775 | 716 | 695 | 551 | 519 | 558 | 728 | 42 | 542 | 20 | <0.01 |
| - | Total BCAA | No Label | | | 262 | 249 | 255 | 245 | 196 | 224 | 255 | 6.2 | 222 | 24 | 0.08 |
| - | Total Aromatic Amino Acids | No Label | | | 133 | 106 | 112 | 91 | 92 | 102 | 117 | 14 | 95 | 6.0 | 0.07 |
| - | Fischer's Ratio | No Label | | | 2.0 | 2.4 | 2.3 | 2.7 | 2.1 | 2.2 | 2.2 | 0.2 | 2.3 | 0.3 | 0.53 |
| - | Total Glu-related Amino Acids | No Label | | | 2,406 | 2,126 | 1,940 | 1,639 | 1,701 | 1,678 | 2,157 | 235 | 1,673 | 31 | <0.05 |
| - | Total Pyr-related Amino Acids | No Label | | | 3,201 | 2,466 | 2,368 | 2,521 | 1,930 | 2,486 | 2,678 | 456 | 2,313 | 331 | 0.32 |
| - | Total Acetyl CoA-related Amino Acids | No Label | | | 289 | 325 | 294 | 216 | 211 | 215 | 302 | 20 | 214 | 2.7 | <0.05 |
| - | Total Fumarate-related Amino Acids | No Label | | | 106 | 87 | 92 | 77 | 75 | 86 | 95 | 9.7 | 79 | 5.9 | 0.07 |
| - | Total Succinyl CoA-related Amino Acids | No Label | | | 233 | 214 | 206 | 195 | 163 | 186 | 218 | 14 | 181 | 16 | <0.05 |
| - | Total Oxaloacetate-related Amino Acids | No Label | | | 377 | 280 | 298 | 358 | 179 | 296 | 318 | 51 | 278 | 91 | 0.54 |
| - | Malate/Asp | No Label | | | 2.8 | 2.6 | 2.4 | 3.0 | 6.6 | 1.9 | 2.6 | 0.2 | 3.9 | 2.5 | 0.47 |
| - | Citrulline/Ornithine | No Label | | | 3.3 | 2.9 | 3.0 | 2.5 | 3.4 | 2.6 | 3.1 | 0.2 | 2.8 | 0.5 | 0.45 |
| - | Glu/2-Oxoglutarate | No Label | | | 1,740 | N.A. | N.A. | N.A. | 64 | N.A. | 1,740 | N.A. | 64 | N.A. | N.A. |
| - | G6P/R5P | No Label | | | 20 | 30 | 32 | 17 | 16 | 19 | 28 | 6.3 | 17 | 1.3 | 0.05 |
| - | SAM/SAH | No Label | | | 10 | 10 | 11 | 9.4 | 13 | 7.4 | 11 | 0.7 | 10 | 3.1 | 0.79 |
| - | Putrescine/Spermidine | No Label | | | 0.2 | 0.2 | 0.2 | 0.09 | 0.12 | 0.14 | 0.2 | 0.02 | 0.12 | 0.02 | <0.05 |

N.D.: Not Detected.

N.A.: Not Available.
