# Supplementary material for: Brown adipocyte-specific knockout of Bmal1 causes mild but significant thermogenesis impairment in mice
Source: Mol Metab. 2021 Mar 3;49:101202. doi: 10.1016/j.molmet.2021.101202 (PMC8042177; doi:10.1016/j.molmet.2021.101202)
Supplement: Supplementary file 3 — Multimedia component 3 [file mmc3.pdf]

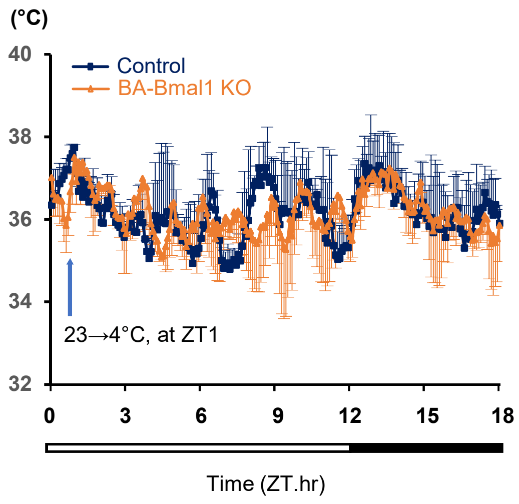

Supplemental Figure 1. BA-Bmal1 KO mice are cold tolerant. Related to Figures 2 and 3.

Core body temperature during cold exposure. The cold exposure (4°C) started at ZT 1. Data are presented as the mean and SD of three mice.

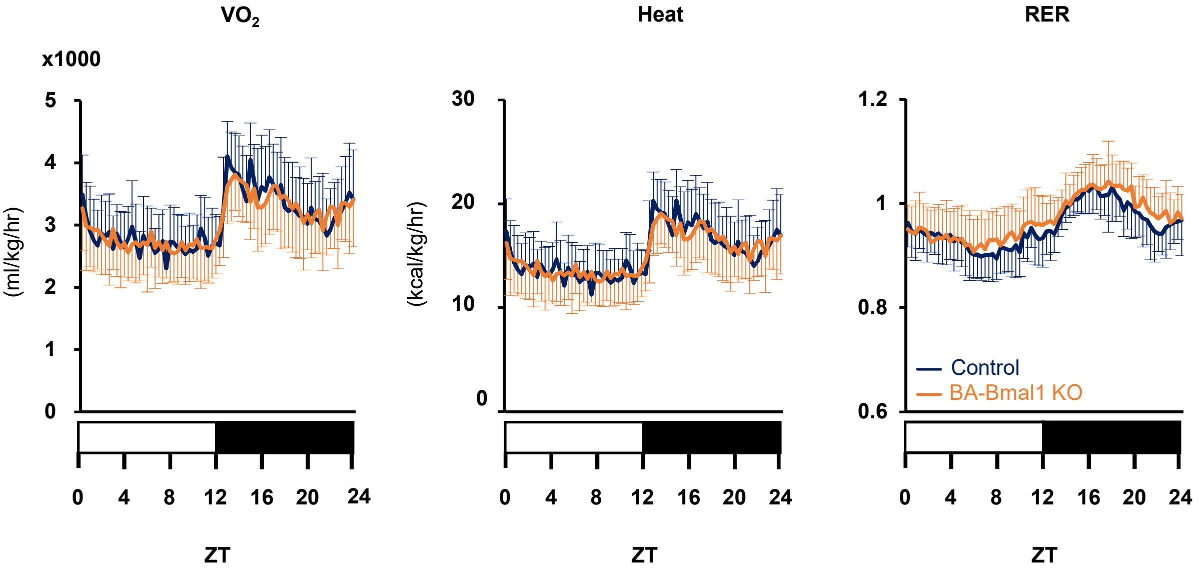

Supplemental Figure 2. Daily profiles of energy expenditure (VO_2 and Heat) and RER. Related to Figure 4A.

Data are presented as the mean and SD of 30 values obtained from five mice.

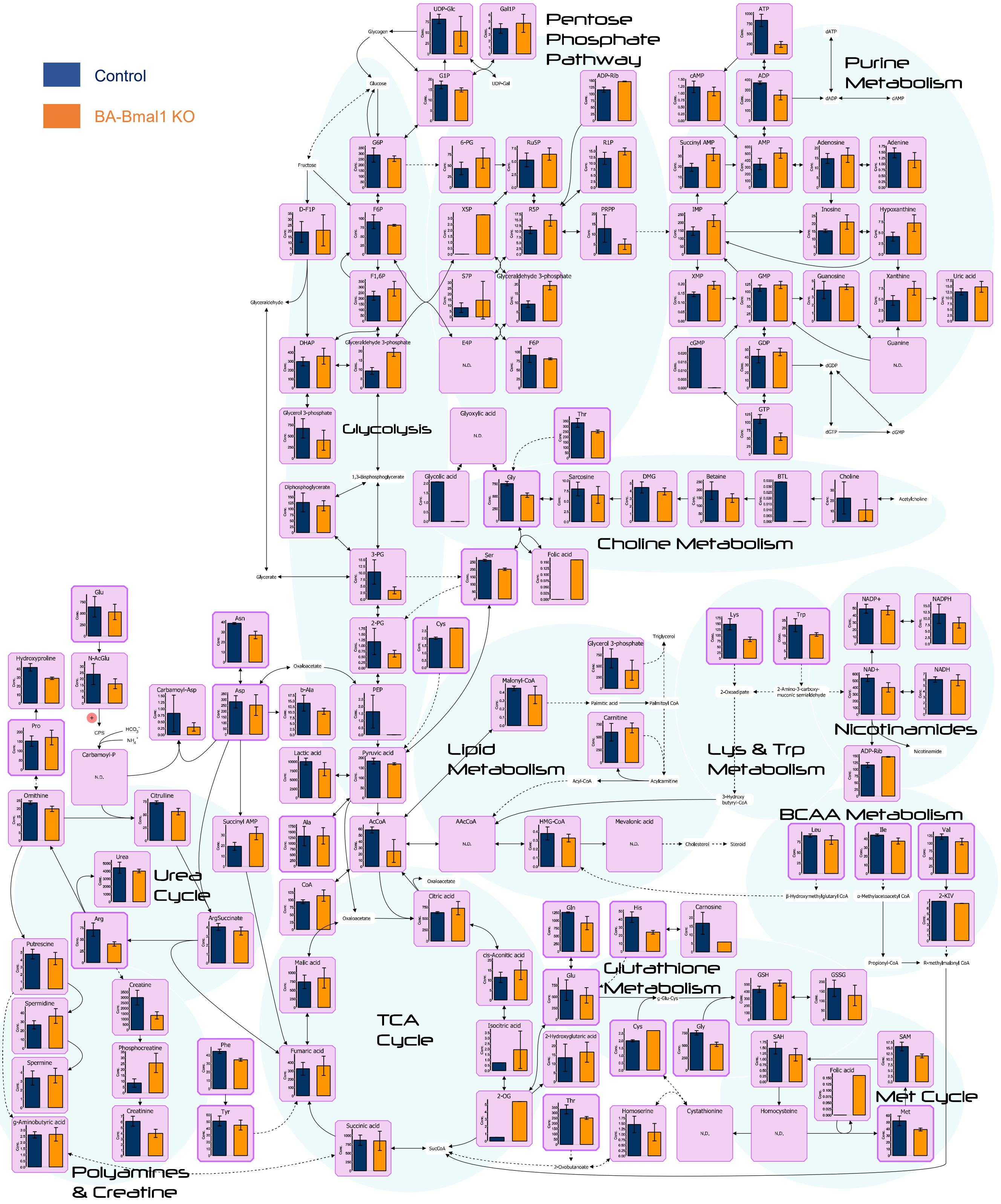

Supplemental Figure 3. Metabolome pathway map of the 116 quantified metabolites. Related to Figure 5.

Data are presented as the mean +/- SD of three mice.

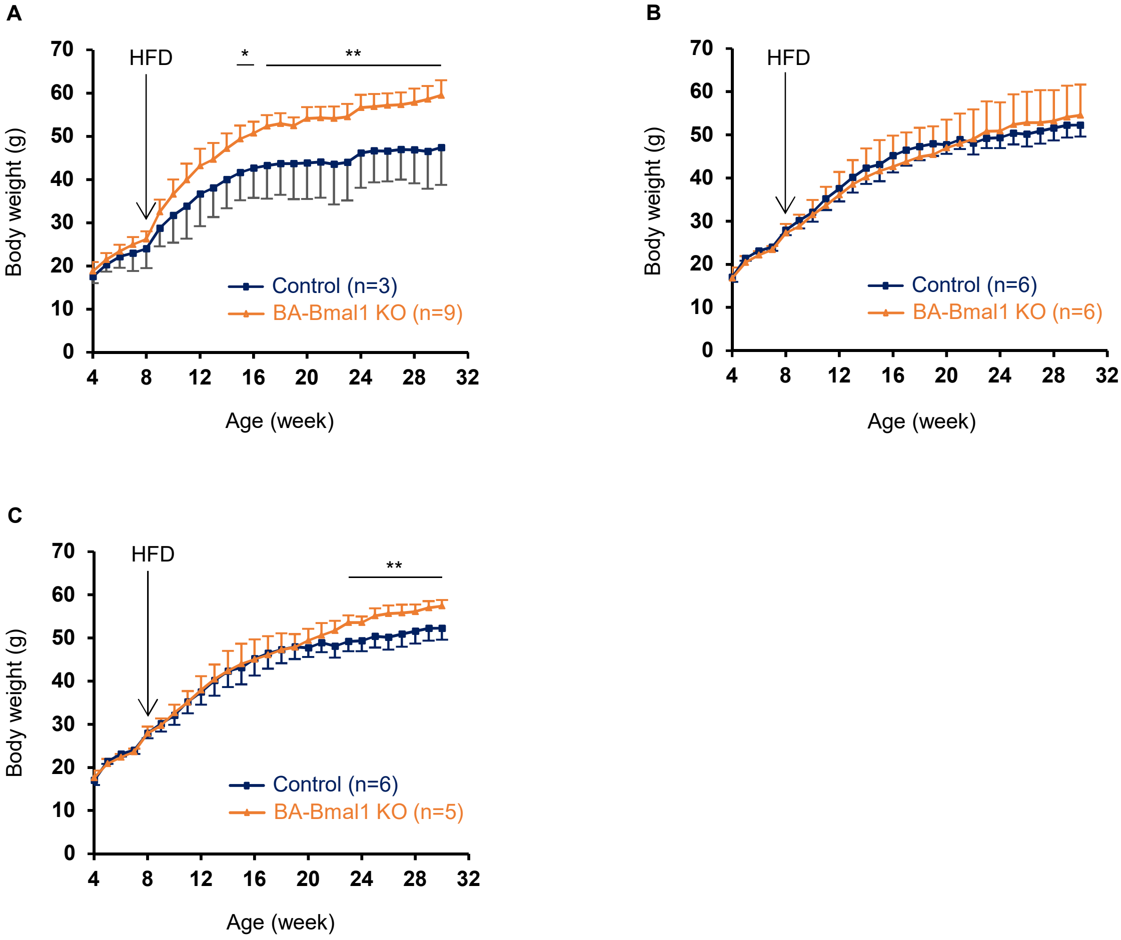

Supplemental Figure 4. Time course of body weight. Related to Figure 6.

(A) Results of the first experiment. BA-Bmal1 KO mice (n = 9) and littermate controls (n = 3) were housed mixedly without determining the genotype. Consequently, the number of BA-Bmal1 KO mice in each cage was 1, 2, 3 and 3, respectively.

(B) Results of the second experiment. Mice (n = 6, each genotype) were separated by their genotypes. One of BA-Bmal1 KO mice was extremely small (body weight, 12.8 g and 40.2 g at 4 and 30 weeks of age, respectively) compared to the other 11 mice (range of body weights, 14.7–19.1 g and 47.7–59.5 g at 4 and 30 weeks of age, respectively).

(C) Results of the second experiment excluding data of the smallest mouse (n = 6 for control, n = 5 for BA-Bmal1 KO).

Mice were housed three animals per cage from 4 weeks of age. High-fat diet feeding was started at 8 weeks of age. Data are presented as the mean and SD. $^*p < 0.05$, $^{**}p < 0.01$ at each time point.
