# Supplementary material for: Brown adipocyte-specific knockout of Bmal1 causes mild but significant thermogenesis impairment in mice
Source: Mol Metab. 2021 Mar 3;49:101202. doi: 10.1016/j.molmet.2021.101202 (PMC8042177; doi:10.1016/j.molmet.2021.101202)
Supplement: Supplementary file 4 — Multimedia component 4 [file mmc4.docx]

**KEY RESOURCES TABLE**

| Reagent or resourse | SOURCE | IDENTIFIER |
| --- | --- | --- |
| Antibodies | | |
| Anti-UCP1 antibody | Abcam | Cat# ab10983; RRID:AB_2241462 |
| Anti-Adipose Triglyceride Lipase antibody | Abcam | Cat# ab109251; RRID:AB_10864772 |
| Anti-CPT1A antibody | Abcam | Cat# ab234111; RRID:AB_2864319 |
| Anti-CPT2 antibody | Abcam | Cat# ab181114; RRID:AB_2687503 |
| Total OXPHOS Rodent WB Antibody Cocktail | Abcam | Cat# ab110413; RRID:AB_2629281 |
| Anti-b-Tubulin antibody | FUJIFILM Wako Pure Chemical | Cat# 010-25043; RRID:AB_2650453 |
| Anti-rabbit IgG, HRP-linked Antibody | Cell Signaling Technology | Cat# 7074; RRID:AB_2099233 |
| Anti-mouse IgG, HRP-linked Antibody | Cell Signaling Technology | Cat# 7076; RRID:AB_330924 |
| Chemicals, Peptides, and Recombinant Proteins | | |
| TRI Reagent | Molecular Research Center | Cat# TR118 |
| Protease inhibitor cocktail | Nacalai Tesque | Cat# 04080 |
| EzWestLumi Plus | ATTO | Cat# 2332637 |
| 4%-Paraformaldehyde Phosphate Buffer Solution | Nacalai Tesque | Cat# 09154-85 |
| Critical Commercial Assays | | |
| RNeasy mini kit | Qiazen | Cat# 74106 |
| High-Capacity cDNA Reverse Transcription Kit | Thermo Fisher Scientific | Cat# 4368814 |
| LabAssay Triglycerides | FUJIFILM Wako Pure Chemical | Cat# 290-63701 |
| LabAssay Cholesterol | FUJIFILM Wako Pure Chemical | Cat# 294-65801 |
| LabAssay NEFA | FUJIFILM Wako Pure Chemical | Cat# 294-63601 |
| Experimental models: Organisms/Strains | | |
| Mouse: *Bmal1*-floxed | Shimba et al., 2011 | RRID:MGI:5613396 |
| Mouse: B6.FVB-Tg(Ucp1-cre)1Evdr/J | Jackson Laboratories | Cat# 024670; RRID:IMSR_JAX:024670 |
| Oligonucleotides | | |
| TaqMan Gene Expression Assays | Thermo Fisher Scientific | See Supplemental Table 1 |
| Software and Algorithms | | |
| Image Studio Lite ver 5.2 | LI-COR | https://www.licor.com/bio/image-studio-lite/ |
| Sleep Sign ver 3.0 | Kissei Comtec | N/A |
| FLIR tools software | FLIR systems | https://www.flir.com/products/flir-tools/ |
| QuPath software | Bankhead et al., 2017 | https://qupath.github.io/ |
| IBM SPSS Statistics ver. 24 | IBM | https://www.ibm.com/products/spss-statistics |
| Other | | |
| Certified diet | Oriental yeast | Cat# CRF-1 |
| High-fat diet | Research Diets | Cat# D12492 |
| ViiA 7 real-time PCR system | Thermo Fisher Scientific | Cat# VIIA7 |
| Glucocard G+ meter | Arkray | Cat# GT-1820 |
| DST nano-T | Star-Oddi | Cat# DST nano-T |
| Supermex | Muromachi Kikai | https://muromachi.com/archives/item/120 |
| FLIR C2 thermal camera | FLIR systems | Cat# C2 |
| Oxymax | Columbus Instruments | Cat# 7540 |
